# Multi‐trait genomic selection for weevil resistance, growth, and wood quality in Norway spruce

**DOI:** 10.1111/eva.12823

**Published:** 2019-06-20

**Authors:** Patrick R. N. Lenz, Simon Nadeau, Marie‐Josée Mottet, Martin Perron, Nathalie Isabel, Jean Beaulieu, Jean Bousquet

**Affiliations:** ^1^ Canadian Wood Fibre Centre Natural Resources Canada Québec Québec Canada; ^2^ Canada Research Chair in Forest Genomics Institute of Integrative Biology and Systems, Centre for Forest Research Université Laval Québec Québec Canada; ^3^ Ministère des Forêts, de la Faune et des Parcs Gouvernement du Québec, Direction de la recherche forestière Québec Québec Canada; ^4^ Laurentian Forestry Centre Natural Resources Canada Québec Québec Canada

**Keywords:** breeding, conifers, index selection, insect resistance, multi‐trait genomic selection, Norway spruce, white pine weevil, wood quality

## Abstract

Plantation‐grown trees have to cope with an increasing pressure of pest and disease in the context of climate change, and breeding approaches using genomics may offer efficient and flexible tools to face this pressure. In the present study, we targeted genetic improvement of resistance of an introduced conifer species in Canada, Norway spruce (*Picea abies* (L.) Karst.), to the native white pine weevil (*Pissodes strobi* Peck). We developed single‐ and multi‐trait genomic selection (GS) models and selection indices considering the relationships between weevil resistance, intrinsic wood quality, and growth traits. Weevil resistance, acoustic velocity as a proxy for mechanical wood stiffness, and average wood density showed moderate‐to‐high heritability and low genotype‐by‐environment interactions. Weevil resistance was genetically positively correlated with tree height, height‐to‐diameter at breast height (DBH) ratio, and acoustic velocity. The accuracy of the different GS models tested (GBLUP, threshold GBLUP, Bayesian ridge regression, BayesCπ) was high and did not differ among each other. Multi‐trait models performed similarly as single‐trait models when all trees were phenotyped. However, when weevil attack data were not available for all trees, weevil resistance was more accurately predicted by integrating genetically correlated growth traits into multi‐trait GS models. A GS index that corresponded to the breeders’ priorities achieved near maximum gains for weevil resistance, acoustic velocity, and height growth, but a small decrease for DBH. The results of this study indicate that it is possible to breed for high‐quality, weevil‐resistant Norway spruce reforestation stock with high accuracy achieved from single‐trait or multi‐trait GS.

## 1 INTRODUCTION

Trees are long‐lived stationary organisms that have to withstand pests and diseases during their lifetime. Host–pest relationships may constantly coevolve over time when organisms share the same environment (Núñez‐Farfán, Fornoni, & Valverde, 2007; Strauss & Agrawal, 1999). However, exotic species may be more exposed to damage by pest insects native to the area where they are introduced (Brockerhoff, Liebhold, & Jactel, 2006) because resistance mechanisms have usually not evolved in response to the selective pressure imposed by native insects, which could potentially compromise the productivity of exotic tree plantations (Branco, Brockerhoff, Castagneyrol, Orazio, & Jactel, 2015). While studies comparing the relative vulnerability of native and exotic conifers to native insects reported mixed results (Fraser & Lawton, 1994; Langström, Lieutier, Hellqvist, & Vouland, 1995; Lombardero, Alonso‐Rodríguez, & Roca‐Posada, 2012; Roques, Auger‐Rozenberg, & Boivin, 2006; Zas, Moreira, & Sampedro, 2011), in the majority of cases, exotic conifers were more damaged than native ones.

Norway spruce (*Picea abies* [L.] Karst) is a conifer native to Scandinavia, Eastern Europe, and the mountainous regions of central Europe. Being the most important commercial softwood for lumber and pulp and paper production in Europe (Hannrup et al., 2004), the species was found to be also highly productive in Eastern North America and one of the most productive conifer in plantation in Quebec, Canada (Thiffault et al., 2003). However, it is highly susceptible to the indigenous white pine weevil (*Pissodes strobi* Peck), a wood‐boring insect found in the temperate and southern boreal forests across the North American continent. Larvae feed on leader shoots, causing dieback; eventually lateral branches take over, causing kink and crooked stems of attacked trees. Besides its principal host eastern white pine (*Pinus strobus* L.), the weevil attacks different native spruces across the North American continent. In Norway spruce, moderate weevil damage leads to significant monetary loss due to stem defects and the resulting losses in lumber volume and quality (Daoust & Mottet, 2006).

The first Norway spruce plantations were established in North America in the 19th century and Canadian early genetic testing efforts of this species date back to the 1920s at the Petawawa National Research Forest (Holst, 1955). Besides the screening for best growing and frost hardy seed sources, it was quickly recognized that breeding efforts needed to include weevil resistance (Holst, 1963). The genetic variation in weevil resistance has been documented in North American spruce species, including Sitka spruce (*Picea stichensis* [Bong.] Carr.) (Alfaro, King, & VanAkker, 2013; Alfaro, VanAkker, Jaquish, & King, 2004; King, 2004) and interior spruce (*Picea glauca* [Moench] Voss × *engelmannii* Parry ex Engelm.; white spruce, Engelmann spruce, and their hybrids) (Alfaro et al., 2004; King, Yanchuk, Kiss, & Alfaro, 1997). Although Norway spruce did not coevolve with the white pine weevil in its native European range, moderate‐to‐high genetic variation and heritability for resistance were reported for this species (Holst, 1955; Mottet, DeBlois, & Perron, 2015). Current Canadian Norway spruce breeding programs are largely based on resistant selections made by the Canadian Forest Service in the past (Daoust & Mottet, 2006). However, selections were made following conventional phenotypic evaluations of mature trees and pedigree‐based approaches, thus requiring many years of testing in genetic experiments. Recent developments in quantitative genomics and breeding such as whole‐genome predictions may help shorten the evaluation stage and breeding cycles (Park, Beaulieu, & Bousquet, 2016).

The application of genomics to forest trees has gained interest to hasten breeding and better understand the genetic control of pest resistance as well as economically important growth and wood quality traits (Plomion, Bousquet, & Kole, 2011). With the common limitation of conventional marker association studies to predict large parts of quantitative genetic variation (e.g., Beaulieu et al., 2011; Gonzalez‐Martinez, Wheeler, Ersoz, Nelson, & Neale, 2007; Porth et al., 2013), efforts in tree breeding have been turning to genomic prediction using dense marker panels. Genomic selection (GS, Box 1) approaches rely on estimating effects of many thousand markers (Meuwissen, Hayes, & Goddard, 2001) or using the realized genomic relationships (***G***) between trees to obtain predictions of genetic values (GBLUPs; VanRaden, 2008). Both GS approaches have been successfully tested in proof‐of‐concept studies in forest trees to predict growth and wood quality, for example, in *Eucalyptus* (Resende, Munoz, et al., 2012; Resende, Resende, et al., 2012), pines (Isik et al., 2016; Resende, Munoz, et al., 2012; Resende, Resende, et al., 2012; Zapata‐Valenzuela, Whetten, Neale, McKeand, & Isik, 2013), and spruces (Beaulieu, Doerksen, Clément, MacKay, & Bousquet, 2014; Beaulieu, Doerksen, MacKay, Rainville, & Bousquet, 2014; Chen et al., 2018; Lenz et al., 2017; Ratcliffe et al., 2015).

Box 1 **Genomic selection: principles and application in tree breeding**

1 Genome‐wide prediction or genomic selection (GS; Meuwissen et al., 2001) relies on simultaneously estimating effects of many thousand markers, with some that are in linkage disequilibrium (LD) with quantitative trait loci (QTL), in order to estimate the genetic merit of an individual. Another GS approach relies on using genetic markers to estimate the realized genomic relationships (***G***) between trees to obtain predictions (GBLUPs) of their genetic value (VanRaden, 2008), as opposed to conventional methods relying on the registered pedigree to make such predictions.

**Figure 1:**
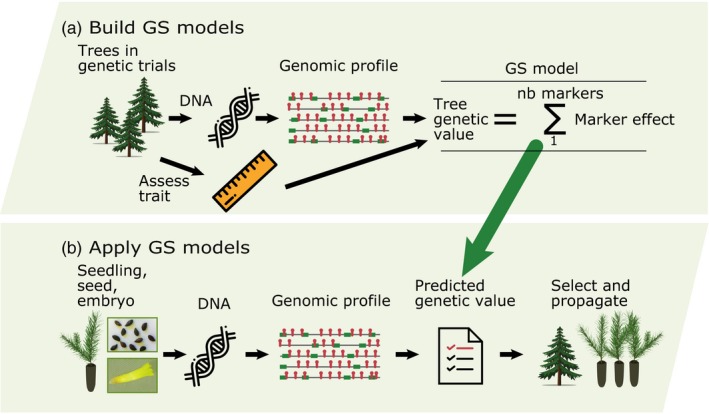
[Box 1] . Genomic selection modeling and integration in tree breeding

**Figure 2:**
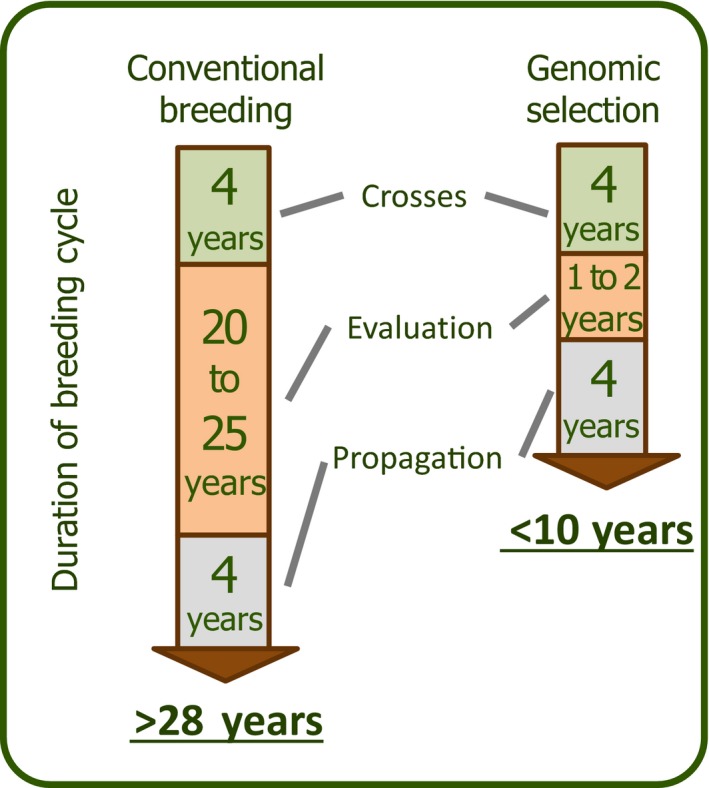
[Box 1] . Estimated time for completing a breeding cycle in (sub‐)boreal conifers such as spruces

Genomic selection models are built using genomic profiles and phenotypic measurements of the same trees in a breeding population (i.e., training population, Figure 1a). Using these models, the prediction of genetic merit can be made based on multilocus genotypes, thus eliminating the need to phenotype and evaluate the performance of candidates for selection (Figure 1b). When predictive models have been validated and are sufficiently accurate, genomic selection can outperform conventional pedigree‐based selection given that genomic profiles from young material (seed, seedling, or embryo) can be obtained to predict genetic values and make selections at a very early stage, thus increasing dramatically genetic gains per unit of time (Figure 2). This is particularly more so for spruces, which can be vegetatively propagated in an efficient fashion from selections made at the juvenile stage (Park et al., 2016). GS is particularly efficient for (sub‐)boreal conifers, where conventional breeding cycles take up to 30 years or longer, largely due to the evaluation stage that can take up to 25 years (Mullin et al., 2011). Hence, under the application of GS, the role of phenotyping is significantly changed and is only needed for model construction and validation. Also, with same genomic profiles, models can be recalibrated with little effort for different traits according to changing breeding priorities. Selection intensity can also be increased by screening large numbers of candidates without phenotyping costs, which is particularly relevant in the context of multi‐trait selection. GS is currently being incorporated into different spruce breeding programs in eastern Canada.

Most of the genomic selection studies in tree species focused on modeling growth and wood traits, which are quantitative traits likely to be controlled by a large number of genes of small effects (Namkoong, Kang, & Brouard, 1988). Despite the fact that many tree breeding programs screen for biotic resistance against pests or disease (Mullin et al., 2011), to our knowledge, there is no study on the accuracy of GS for insect resistance in trees. One study tested different GS models to predict resistance to a pathogen in loblolly pine (*Pinus taeda* L.) (Resende, Munoz, et al., 2012; Resende, Resende, et al., 2012). The authors reported that fusiform rust resistance was likely controlled by a few genes of large effects since it was best predicted with Bayesian regression models that allowed for different variance of marker effects. Not only is the genetic architecture of the trait an important consideration in the choice of GS model, but one must also deal with the nature of the phenotypic data. Screening data for pest resistance is most often qualitative or semiquantitative, which needs appropriate statistical approaches that can handle non‐normality of modeling errors. Thus, there is an opportunity for testing different GS approaches with weevil resistance data in Norway spruce, for example the Bayesian generalized linear regression models that support binary or ordinal data (Perez & de los Campos, 2014) or the threshold GBLUP model developed for ordinal data (Montesinos‐López et al., 2015).

In practice, breeders generally need to consider multiple traits simultaneously in their selections for improved genetic stock. Besides reducing insect attack, improving growth and wood volume are major goals for Norway spruce, as it is for several plantation‐grown conifers (Mullin et al., 2011). However, reduction of wood quality was observed in the past under selection for accelerated growth (Chen et al., 2014; Lenz, Cloutier, MacKay, & Beaulieu, 2010). Therefore, important wood traits for mechanical applications such as wood stiffness should be considered when performing selections (Lenz, Auty, Achim, Beaulieu, & Mackay, 2013). Mottet et al. (2015) concluded that selection for weevil resistance would not reduce height growth, but could affect diameter and therefore volume. However, the genetic relationships between weevil resistance and intrinsic wood properties such as wood density and stiffness have never been investigated. Thus, there is a need to understand the genetic correlations between weevil resistance, wood quality, and growth traits and to look at the possibility to combine them in a multi‐trait genomic selection framework.

Multivariate genomic selection models can improve the accuracy of predictions by taking advantage of the genetic correlations between traits (Calus & Veerkamp, 2011). This ability is especially advantageous for prediction of traits that are costly or difficult to measure by conventional means on a large number of candidate trees, such as weevil resistance or wood quality, by using available correlated indicator traits. Simulation studies showed that multi‐trait models can increase the accuracy of a target trait of low heritability when it is modeled together with genetically correlated indicator traits harboring high heritability (Guo et al., 2014; Jia & Jannink, 2012). In addition, the benefits of multi‐trait GS are increased for target traits with scarce phenotypic records when they are coupled with intensively phenotyped indicator traits (Guo et al., 2014; Jia & Jannink, 2012; Schulthess et al., 2016). Few studies have applied multi‐trait genomic selection methods to real plant breeding datasets (e.g., Bao, Kurle, Anderson, & Young, 2015; Fernandes, Dias, Ferreira, & Brown, 2018; Schulthess et al., 2016). In tree species, the accuracy of multi‐trait versus single‐trait GS models was only tested on a few traits using bivariate models in loblolly pine (Cheng, Kizilkaya, Zeng, Garrick, & Fernando, 2018; Jia & Jannink, 2012) and in *Eucalyptus* (Cappa et al., 2018), and these studies did not investigate the effects of missing phenotypic data. Given the observed positive genetic correlations between weevil resistance and height growth (Mottet et al., 2015), and the possible correlations with wood quality traits, multi‐trait GS models could improve the accuracy of predictions for traits that are difficult and expensive to assess on a large number of trees.

Once accurate genomic‐estimated breeding values for each trait have been obtained from either single‐trait or multi‐trait models, it is possible to combine them into a selection index (SI; Hazel, 1943) and to rank individuals based on their overall performance across all traits. This strategy is especially useful in the presence of negative genetic correlations in order to select material that achieve a good balance in their performance for all traits of interest. In addition, GS is especially well suited to allow identifying correlation breakers in sufficient number, given the higher selection intensities that can be achieved by screening larger numbers of candidates than with conventional methods (Park et al., 2016). Hence, multi‐trait genomic selection models and index selection are two different tools that can be combined to improve accuracies of breeding values and optimize genetic gains, respectively, in a multi‐trait breeding program.

Here, we present a comprehensive genetic study of weevil resistance in Norway spruce and its relationships with growth and wood quality traits in the context of establishing a multi‐trait genomic selection breeding program. Our objectives were to (a) better understand the genetic relationships between weevil attack and other growth and wood traits, in particular intrinsic wood quality traits; (b) evaluate the performance of different single‐trait genomic selection models, especially for weevil resistance; (c) test the performance of multi‐trait genomic selection models for predicting a target trait (weevil resistance or wood quality) when coupled with genetically correlated indicator traits (e.g., height growth); and (d) develop multi‐trait genomic selection indices for the production of high‐quality and weevil‐resistant seedling stock in Norway spruce.

## 2 MATERIAL AND METHODS

### 2.1 Genetic material and phenotyping

**Figure 3:**
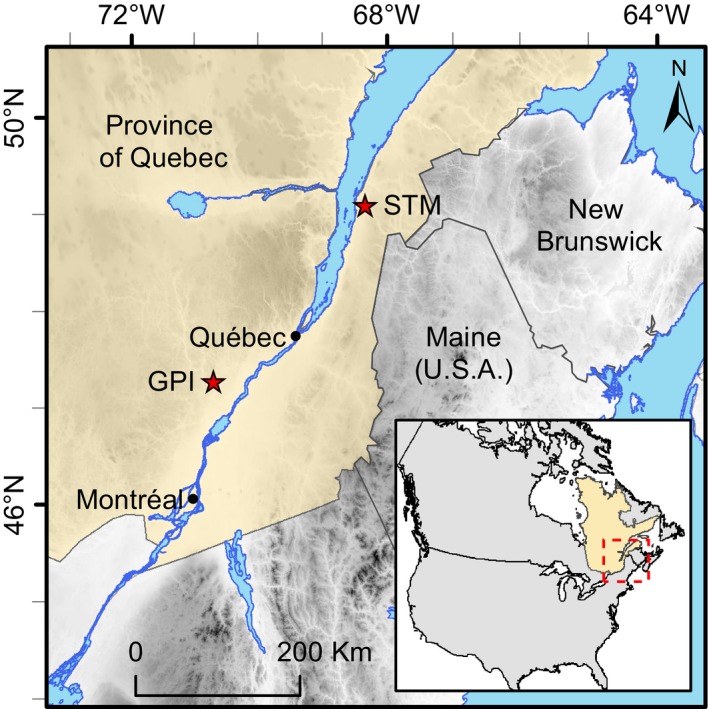
Location of the test sites Saint‐Modeste (STM) and Grandes‐Piles (GPI) in the province of Québec, Canada

The data analyzed in this study are a subset of a larger breeding population derived from a partial diallel mating design (see Mottet et al., 2015 for more details). We focused our phenotyping and genotyping efforts on the trees planted on two sites affected by white pine weevil in Quebec, that is, Saint‐Modeste (47.85°N; 69.38°W; elevation: 140 m; abbreviated STM) and Grandes‐Piles (46.68°N; 72.68°W; elevation: 150 m; abbreviated GPI), respectively, located in the balsam fir–yellow birch and the sugar maple–yellow birch bioclimatic domains (Figure 3). Both tests were set up in year 2000. The Grandes‐Piles plantation was heavily affected by weevils with ~70% of the trees attacked at least once by age 16, while the Saint‐Modeste plantation was moderately affected (~47% of the trees attacked by age 16). To develop genomic selection models for weevil resistance, we selected 40 full‐sib families (35 parents), 20 of which were rated resistant and 20 rated susceptible based on weevil attack surveys at ages 10 and 15 on these two sites. A total of 726 trees (14 to 20 per family, mean = 17.85) were sampled. Each parent was crossed on average 2.3 times (Figure S1). The two trials were established according to a randomized complete block design, each with five blocks and three‐tree row plots for each family (tree spacing: 2.5 m × 2 m). Because of tree mortality, not all families were represented in every block (i.e., incomplete block design).

Tree height (Height_15_) and diameter at breast height (DBH_15_) were measured at age 15. The height‐to‐diameter ratio (Height_15_/DBH_15_) was calculated as a proxy of stem taper. Three wood quality traits related to mechanical properties were assessed: average wood density and cellulose microfibril angle were determined from wood increment cores using X‐ray densitometry and diffractometry at age 15 (Density_15_ and MFA_15_) as described in Lenz et al. (2017); acoustic velocity was measured at age 16 (Velocity_16_) with the ST300 Hitman tool. Acoustic velocity is a proxy for wood stiffness or modulus of elasticity measured at standing trees (Chen et al., 2015; Desponts, Perron, & DeBlois, 2017; Lenz et al., 2013).

For each of two surveys at ages 10 and 15, the presence/absence of weevil damage in the current year (coded 0/1) and in previous years (coded 0/1) were recorded. Current year damage was detected by inspecting the terminal shoot for emergence holes or death of the leader shoot. Weevil damage in previous years was visible by forks, curves, bayonets, or multiple stems. We calculated the cumulative number of attacks (CWA) as:

(1) $$CWA=WA_{previous10}+WA_{current10}+WA_{11-14}+WA_{current15}$$

where WA_current10_ and WA_current15_ are the presence/absence of attack at age 10 and 15, respectively; WA_previous10_ is the presence/absence of attack prior to age 10; and WA_11–14_ is the presence/absence of attack between ages 11 and 14. The variable WA_11–14_, which was calculated to avoid double‐counting previous attacks, was equal to 1 (presence) if there was an attack prior to age 15, but no attack at age 10 or prior to age 10. The resulting CWA variable is ordinal (ordered categories of 0, 1, 2, and 3 weevil attacks). Table 1 and Figure S2 provide the summary statistics and violin plots, respectively, for the traits assessed in this study. Figure S3 shows the spatial distribution of CWA values in the test sites.

**Table 1:** Phenotypic means, standard deviations (*SD*), and coefficients of variation (CV) for each site and across sites for the 714 trees retained for analyses

Trait^a^	Units	GPI^b^ (*n* = 388)			STM^b^ (*n* = 326)			Across sites (*n* = 714)		
		Mean	*SD*	CV (%)	Mean	*SD*	CV (%)	Mean	*SD*	CV (%)
Velocity_16_	km/s	3.71	0.33	8.96	3.69	0.31	8.54	3.70	0.32	8.77
Density_15_	kg/m^3^	344.78	24.77	7.19	395.45	29.30	7.41	367.91	36.91	10.03
MFA_15_	degrees	10.98	4.59	41.81	13.22	6.47	48.96	12.01	5.64	46.98
DBH_15_	mm	148.05	20.08	13.56	106.73	20.49	19.19	129.19	28.89	22.36
Height_15_	cm	908.09	128.12	14.11	788.90	111.64	14.15	853.67	134.61	15.77
Height_15_/DBH_15_	—	62.08	10.01	16.13	75.46	11.61	15.39	68.19	12.67	18.57
CWA	Number of attacks	0.99	0.80	80.79	0.63	0.76	121.65	0.83	0.81	97.46

a Measured traits in descending order are acoustic velocity at age 16 as a proxy for wood stiffness, average wood density at age 15, microfibril angle at age 15, diameter at breast height at age 15, tree height at age 15, the height‐to‐diameter ratio at age 15, and the cumulative number of weevil attacks.

b Experimental sites Grandes‐Piles (GPI) and Saint‐Modeste (STM).

### 2.2 SNP genotyping

The 726 trees were genotyped using an Infinium iSelect SNP array (Illumina), which was assembled from a catalog of high‐confidence gene SNPs obtained from exome capture and sequencing (Azaiez et al., 2018). The genotyping reproducibility rate was high (99.94%), as estimated from two positive controls replicated on each genotyping plate. From 5,660 successfully manufactured SNPs representing as many distinct gene loci well distributed over the 12 spruce linkage groups (Pavy et al., 2017), we retained a total of 3,914 SNPs with call rate ≥90% (average call rate of 99.6%), minor allele frequency (MAF) ≥0.005, and a fixation index |*F*_IS_| < 0.50. SNPs were well distributed across MAF classes with 86% of the SNPs with MAF ≥0.05 (Figure S4). Missing genotypes (only 0.4% of genotypes) were imputed using a *k*‐nearest neighbor method based on linkage disequilibrium (LD‐kNNi) with the software LinkImpute (Money et al., 2015). The software estimated an accuracy of 0.83 for imputed genotypes by randomly masking genotypes.

### 2.3 Relationship matrices and pedigree verification

All analyses were performed in the R v.3.3.1 environment (R Core Team, 2016). A pedigree‐based relationship matrix (***A***) and its inverse were first computed based on the registered pedigree information using the function “asreml.Ainverse” of the R package ASReml‐R v.3.0 (Butler, Cullis, Gilmour, & Gogel, 2007). For use in genomic selection (GS) models, the realized genomic relationship matrix (***G***, Figure S5) was computed from the marker data with the “A.mat” function of the R package rrBLUP (Endelman & Jannink, 2012) with the default options, which was equivalent to the formula described by VanRaden (2008). A comparison of the ***A*** and ***G*** matrices revealed 11 “misclassified” trees that presumably did not belong to their expected cross. In addition, one tree was deemed an outlier because it had abnormally high average wood density (Density_15_), after checking model residuals (see Equation (2) below). After removing misclassified and outlier trees, 714 trees genotyped on 3,914 SNPs were used in subsequent analyses. The resulting ***A*** and ***G*** matrices were highly correlated with a Pearson *r* = 0.94 (Figure S6). Large values of ***G*** within each class of ***A*** are mostly due to two inbred families (Figure S7).

### 2.4 Heritability and genotype‐by‐environment interactions

First, variance components, heritability, and breeding values were estimated using the conventional pedigree‐based (ABLUP) or the genomic‐based (GBLUP) individual‐tree mixed models (the so‐called “animal model”) in ASReml‐R v.3.0:

(2) $$y=\mu +Xs+Z_{1}b\left(s\right)+Z_{2}a+Z_{3}sa+e$$

where ***y*** is the phenotype; *μ* is the overall mean; ***s*** is the fixed site effect; ***b***(***s***) is the random effect of block within site, with $b\left(s\right)∼N\left(0,\sigma_{b}^{2}I_{b}\right)$; ***a*** is the random additive genetic effect, with $a∼N\left(0,\sigma_{a}^{2}A\right)$; ***sa*** is the random interaction of site with additive genetic effects, with $sa∼N(0,\sigma_{\mathrm{sa}}^{2}I_{s}⊗A$); and ***e*** is the residual term, assuming homogeneity across sites with $e∼N(0,\sigma_{e}^{2}I_{e}$). For the ABLUP method, the matrix ***A*** is the pedigree‐based relationship matrix, which was replaced by the realized genomic relationship matrix ***G*** for the GBLUP method ($a∼N\left(0,\sigma_{a}^{2}G\right)$ and $sa∼N(0,\sigma_{\mathrm{sa}}^{2}I_{s}⊗G$)). The ***X*** and ***Z*** matrices are incidence matrices of their corresponding effects, and ***I_x_*** is an identity matrix of its proper dimension. The symbol ⊗ refers to the Kronecker product. To test the hypothesis of greater than zero variance for each effect (*H*_0_: *σ* ^2^ = 0; *H*_1_: *σ* ^2^ > 0), we performed a likelihood‐ratio test with one degree of freedom between the full model in Equation (2) and a reduced model without the effect to be tested. The dominance effect was not included in models because it was not significant for all traits under study (Table S1), and, according to BIC, the fit of models including dominance was similar or worse than the models including the additive effect only (Table S2). We compared model (1) with a model fitting a different residual variance for each site and found that the latter model was slightly better for three out of the seven traits studied (Table S3). However, the breeding values between both approaches were highly correlated for all traits, with *r* > 0.99. Thus, we opted for the simpler model in Equation (2) to keep ABLUP and GBLUP models comparable with marker‐based genomic selection models. Finally, inspection of the residuals from Equation (2) versus the distance in X and Y in the trials showed no detectable spatial patterns (not shown).

Narrow‐sense individual heritability was estimated as:

(3) $${\hat{h}}_{\mathrm{ind}}^{2}={\hat{\sigma }}_{a}^{2}/\left({\hat{\sigma }}_{a}^{2}+{\hat{\sigma }}_{\mathrm{sa}}^{2}+{\hat{\sigma }}_{e}^{2}\right)$$

The size of genotype‐by‐environment interaction (GxE), or type‐B correlation (${\hat{r}}_{B}$), was estimated as:

(4) $${\hat{r}}_{B}={\hat{\sigma }}_{a}^{2}/\left({\hat{\sigma }}_{a}^{2}+{\hat{\sigma }}_{\mathrm{sa}}^{2}\right)$$

Standard errors of heritability and type‐B correlation estimates were obtained using the delta method (pin function from the R package nadiv; Wolak, 2012).

### 2.5 Correlations between weevil resistance, wood quality, and growth traits

To estimate single‐site phenotypic and genetic correlations between traits, bivariate models were run for all pairs of traits in ASReml. Bivariate models were run for each site separately because of convergence problems of the multi‐site model due to large GxE for some traits (e.g., DBH_15_, see Results). The following model was fitted:

(5) $$\left[\begin{array}{c} y_{i}\\ y_{j} \end{array}\right]=Xt+Z_{1}b\left(t\right)+Z_{2}a\left(t\right)+e$$

where ***y_i_*** and ***y_j_*** are the stacked vectors of phenotypic observations for trait *i* and trait *j,* respectively; ***t*** is the vector of fixed effects of traits (*i.e.,* the grand mean for each trait); ***b***(***t***) is the random effect of block nested within trait, with $b\left(t\right)∼N\left(0,I_{b}⊗V_{B}\right)$; ***a***(***t***) is the random additive effect within trait, with $a\left(t\right)∼N\left(0,A⊗V_{A}\right)$; and ***e*** is the residual error, with $e∼N\left(0,I_{e}⊗V_{R}\right).$ The matrix ***A*** (ABLUP) was replaced by the realized genomic relationship matrix (***G***) for the GBLUP method. The matrices ***V_B_***, ***V_A_***, and ***V_R_*** are 2 x 2 variance–covariance matrices defined by the correlation of effects between traits (*r_b_*, *r_a_*, and *r_e_*, respectively) and unique variances for each trait (i.e., CORGH in ASReml). To facilitate convergence, we provided starting values for the variance components in ***V_B_***, ***V_A_***, and ***V_R_*** matrices that were taken from the results of the single‐trait models (Equation (2), Table S4). For *r_b_*, *r_a_*, and *r_e_*, the starting value was set to 0 (no correlation). The genetic correlation between traits was directly provided by the estimated parameter ${\hat{r}}_{a}$ and the phenotypic correlation was calculated as:

(6) $${\hat{r}}_{P}=\frac{COV{\left(i,j\right)}_{p}}{\sqrt{{\hat{\sigma }}_{\mathrm{pi}}^{2}{\hat{\sigma }}_{\mathrm{pj}}^{2}}}=\frac{{\hat{r}}_{b}\sqrt{{\hat{\sigma }}_{\mathrm{bi}}^{2}{\hat{\sigma }}_{\mathrm{bj}}^{2}}+{\hat{r}}_{a}\sqrt{{\hat{\sigma }}_{\mathrm{ai}}^{2}{\hat{\sigma }}_{\mathrm{aj}}^{2}}+{\hat{r}}_{e}\sqrt{{\hat{\sigma }}_{\mathrm{ei}}^{2}{\hat{\sigma }}_{\mathrm{ej}}^{2}}}{\sqrt{\left({\hat{\sigma }}_{\mathrm{bi}}^{2}+{\hat{\sigma }}_{\mathrm{ai}}^{2}+{\hat{\sigma }}_{\mathrm{ei}}^{2}\right)\left({\hat{\sigma }}_{\mathrm{bj}}^{2}+{\hat{\sigma }}_{\mathrm{aj}}^{2}+{\hat{\sigma }}_{\mathrm{ej}}^{2}\right)}}$$

where COV(*i,j*)*_p_* is the phenotypic covariance between traits, and ${\hat{\sigma }}_{\mathrm{pi}}^{2}$, ${\hat{\sigma }}_{\mathrm{bi}}^{2}$, ${\hat{\sigma }}_{\mathrm{ai}}^{2}$, and ${\hat{\sigma }}_{\mathrm{ei}}^{2}$ are the estimated phenotypic, block, additive, and residual variance of trait *i* (same for trait *j*), respectively. The significance of the genetic correlation (*H*_0_: *r_a_* = 0; *H*_1_: *r_a_* ≠ 0) was tested by performing a likelihood‐ratio test with one degree of freedom between the full model in Equation (5) and a reduced model assuming *r_a_* = 0 (*i.e.*, a diagonal ***V_A_*** matrix). The significance of the phenotypic correlation (*H*_0_: *r_p_* = 0; *H*_1_: *r_p_* ≠ 0) was tested by performing a likelihood‐ratio test with three degrees of freedom between the full model in Equation (5) and a reduced model assuming no correlation between traits (*r_b_* = 0, *r_a_* = 0, and *r_e_* = 0). The single‐site heritability of trait *i* was given by:

(7) $${\hat{h}}_{ind\;ss}^{2}={\hat{\sigma }}_{\mathrm{ai}}^{2}/\left({\hat{\sigma }}_{\mathrm{ai}}^{2}+{\hat{\sigma }}_{\mathrm{ei}}^{2}\right)$$

### 2.6 Single‐trait genomic selection models

We evaluated four single‐trait GS methods: GBLUP, Bayesian ridge regression (BRR), BayesCπ, and, in addition for CWA, threshold GBLUP (TGBLUP) developed for ordinal traits. GBLUP was implemented as described in Equation (2) in ASReml. The ***G*** matrix describes the realized genomic relationships between trees, which better account for within‐family Mendelian sampling, as well as deeper (unknown) pedigree relationships. GBLUP relies on the “infinitesimal” model of quantitative genetics, assuming that the genetic control of complex traits is equally distributed across many (infinite) loci with small effects (Falconer & Mackay, 1996). The GBLUP model assumed that residuals are normally distributed for all traits. For the trait CWA, we tested the threshold GBLUP model (TGBLUP) for ordinal data, as described by Montesinos‐López et al. (2015). To implement TGBLUP, the same model as in Equation (2) was fitted, but the response type was set to “ordinal” (probit link function) in the BGLR R package v.1.0.5 (de los Campos, & Pérez‐Rodríguez, 2016). Conventional‐estimated breeding values (EBVs) of individual trees for the ABLUP method or the genomic‐estimated breeding values (GEBVs) for the GBLUP and TGBLUP methods were obtained from the best linear unbiased predictions (BLUPs) of the random additive effect (***a***).

BRR and BayesCπ are from a different class of models, in which marker effects are estimated and used to predict breeding values based on tree genotypes. We fitted the following Bayesian model in the BGLR package:

(8) $$y=\mu +Xs+Z_{1}b\left(s\right)+Z_{2}a_{m}+e$$

where ***y*** is the phenotype; *μ* is the overall mean; *s* is the fixed site effect (*i.e.,* modeled with a flat prior); ***b***(***s***) is the random effect of block within site, with $b\left(s\right)∼N\left(0,\sigma_{b}^{2}I_{b}\right)$; ***a_m_*** is the random additive effect of markers; and ***e*** is the residual term, with $e∼N(0,\sigma_{e}^{2}I_{e}$). Note that compared with ABLUP and GBLUP models, the genotype‐by‐environment interaction (GxE) component was not fitted in BRR and BayesCπ models, thus assuming that marker effects were stable across environments. To account for different distributions of marker effects, the prior for ***a_m_*** changes depending on the method (see Appendix S1 for a full description). Briefly, the method BRR is a Bayesian version of ridge regression, in which marker effects are normally distributed (i.e., Gaussian prior) and have identical variance ($a_{m}∼N(0,\sigma_{m}^{2}I_{m}$)). In BRR, all markers have a nonzero effect, and so this method is appropriate for traits controlled by a large number of genes with small effects. In contrast, the method BayesCπ takes into account that only a proportion *π* of markers have an effect, while a proportion (1 − π) of marker effects are shrunk toward zero (Habier, Fernando, Kizilkaya, & Garrick, 2011). For the BRR and BayesCπ methods, the response type was set to “ordinal” (probit link function) for the trait CWA_, _and to “gaussian” for all other traits. BGLR was run for 50,000 iterations and a thinning interval of 20, with the first 15,000 iterations discarded as a burn‐in. Genomic‐estimated breeding values (GEBVs) were obtained by summing over the effects of all markers, with ${\mathrm{GEBV}}_{i}=\sum_{j=1}^{m}{Z\text{'}}_{\mathrm{ij}}{\hat{a}}_{j}$, where ${\hat{a}}_{j}$ is the estimated effect of the *j* ^th^ marker, and $Z_{\mathrm{ij}}^{\prime }$ is an indicator of the genotype of individual *i* at the *j^th^* marker.

### 2.7 Multi‐trait genomic selection models

Genomic selection models that incorporated the information of multiple correlated traits into a single analysis were evaluated using GBLUP multivariate models in ASReml. To facilitate the convergence of multivariate models, the modeling was done in two steps. First, phenotypes were adjusted for block and site effects (y*) by taking the residuals (***e***) of a model that included a fixed site effect (***s***) and a random block within site effect (***b***(***s***)): $y=\mu +Xs+Zb\left(s\right)+e$. After adjusting phenotypes, the portion of GxE due to rank changes in different sites remains, but the “level‐of‐expression GxE” (i.e., spread of breeding values across environments) is controlled for (Li, Suontama, Burdon, & Dungey, 2017). Second, a multivariate model with *p* traits was fitted:

(9) $$\left[\begin{array}{c} {y^{*}}_{i}\\ \cdots \\ {y^{*}}_{p} \end{array}\right]=Xt+Za\left(t\right)+e$$

where ***y*** ^*^ ***_i_*** are the stacked vectors of adjusted phenotypes from trait *i* to trait *p*; ***t*** is the vector of fixed effects of traits (*i.e.,* the grand mean for each trait); ***a***(***t***) is the random additive effect within trait, with $a\left(t\right)∼N\left(0,G⊗V_{A}\right)$; and *e* is the residual error, with $e∼N\left(0,I_{e}⊗V_{R}\right)$. The matrices ***V_A_***, and ***V_R_*** are *p* × *p* variance–covariance matrices, defined by correlations between all pairs of traits and unique variances for each trait. GxE (as rank‐changes interaction) was not fitted to simplify the model and facilitate convergence. We provided starting values for the variance components in ***V_A_*** and ***V_R_*** matrices that were taken from the results of the single‐trait models. We obtained GEBVs of individual trees for each trait separately from the BLUPs of the random additive effect (***a***) within trait.

We evaluated the performance of multi‐trait GS models for predicting a target trait, when coupled with genetically correlated indicator traits. We chose three target traits, the cumulative number of weevil attacks (CWA), average wood density at age 15 (Density_15_), and microfibril angle at age 15 (MFA_15_), for which measurements are difficult or costly to obtain for a large number of candidate trees. For each target trait, four multi‐trait GS model was tested: (a) a two‐trait model including one of the target traits and Height_15_ as an indicator trait; (b) a two‐trait model with one of the target traits and Height_15_/DBH_15 _ratio; (c) a two‐trait model with one of the target traits and Velocity_16_; and (d) a three‐trait model with one of the target traits, Height_15_/DBH_15_ ratio, and Velocity_16_. To simulate a situation where the target trait was only measured for a smaller subset of the trees, the percentage of missing data in the training set (see below) was varied from 0% to 90% by randomly adding missing values to the phenotypes of the target trait. We repeated this process 100 times for each trait and level of random missing values in cross‐validation (see below).

### 2.8 Cross‐validation and estimation of accuracy

The prediction accuracy of ABLUP, GBLUP (single and multi‐trait), TGBLUP, BRR, and BayesCπ models’ predictions was tested using a tenfold cross‐validation (CV) scheme combining data across sites as in Beaulieu, Doerksen, Clément, et al. (2014) and Lenz et al. (2017). The full set of individual trees was randomly split into tenfold, each containing ~10% of the trees from each family. For each round of CV, ninefold (~642 trees or 90%) was used in model training, which was used to predict the breeding values for the remaining fold (~71 validation trees or 10%). This tenfold CV was repeated ten times, for a total of 100 models for each trait.

The predictive ability (PA) of the models was evaluated as the Pearson correlation coefficient between the predicted breeding values of the validation trees and the adjusted phenotypes (y*) for block and site effects. The predictive accuracy (PACC) of models was estimated from PA as $\text{PACC}=\text{PA}/\sqrt{{\hat{h}}_{\mathrm{ind}}^{2}}$(Dekkers, 2007; Legarra, Robert‐Granie, Manfredi, & Elsen, 2008). For all the methods tested (ABLUP, GBLUP, TGBLUP, BRR, BayesCπ, and multi‐trait GBLUP), we used the ${\hat{h}}_{\mathrm{ind}}^{2}$ estimated from the GBLUP model (Equations 2 and 3) as our best estimate of heritability for the calculation of predictive accuracy (PACC). PA and PACC were calculated within each fold to avoid including a fold effect, then averaged across folds and repetitions to obtain standard errors (Isik, Holland, & Maltecca, 2017).

For multi‐trait models, the prediction ability (PA) and prediction accuracy (PACC) of multi‐trait GBLUP models were compared with the equivalent single‐trait GBLUP models using adjusted phenotypes as a response variable (see Appendix S2).

### 2.9 Genetic gains and multi‐trait selection indices

Expected genetic gains from single‐trait selection were calculated as the mean estimated breeding value of the top 5% trees for each trait separately, as estimated from the single‐trait ABLUP (EBVs) or GBLUP (GEBVs) analysis (Equation 2). These estimated gains represented the maximum possible gain for each trait. To estimate genetic gains in a multi‐trait selection context, we combined four traits of economic interest into a SI as follows:

(10) $$\mathrm{SI}=w_{1}{Height}_{15EBV}-w_{2}{CWA}_{6.15EBV}+w_{3}{Velocity}_{16EBV}+w_{4}{Density}_{15EBV}$$

where ${\mathrm{Height}}_{15\mathrm{EBV}}$, ${\mathrm{CWA}}_{6.15\mathrm{EBV}}$, ${\mathrm{Velocity}}_{16\mathrm{EBV}}$, and ${\mathrm{Density}}_{15\mathrm{EBV}}$ are the BLUP estimated breeding values from the single‐trait ABLUP or GBLUP analysis (Equation 2) for the corresponding trait; and *w_i_* are the relative weight given to each trait, with the restrictions:

$0\leq w_{i}\leq 1$ for all traits, and
$w_{1}+w_{2}+w_{3}+w_{4}=1$.

We assigned a negative sign to CWA since a decrease in the value of this trait (fewer weevil attacks) represents an improvement. Breeding values for each trait were scaled to a variance of one (already centered) prior to SI calculations. DBH_15_ is an economically important trait, but was excluded from the SI scenarios because its heritability was not significantly different from zero (see results). The four weight coefficients were varied between 0 and 1 by intervals of 0.05, resulting in 1,771 different selection indices. For each SI, trees were ranked according to decreasing values of the index (***I***) and the top 5% trees were selected to calculate the expected genetic gain. For each trait, the relative genetic gain (%) was calculated as the ratio of the expected gain to the maximum possible gain from single‐trait selection. We present, a first SI scenario (SI‐1) that corresponded to the priorities for the Norway spruce breeding program in Québec, which put more emphasis on weevil resistance (*w*_2_ = 0.6), followed by growth, represented here by height growth (*w*_1_ = 0.3), and acoustic velocity (*w*_3_ = 0.1). We chose to present two further SIs that maximized the total relative gain of the following traits: Height_15_, CWA, and Velocity_16 _(SI‐2) and in the other case Height_15_, CWA, Velocity_16_, and Density_15_ (SI‐3).

## 3 RESULTS

### 3.1 Heritability and genotype‐by‐environment interaction

In the across‐site analyses, heritability ranged from 0 to 0.47 using the ABLUP method and from 0 to 0.29 using GBLUP (Table 2). Details of estimated variance components are in Table S4. We found moderate‐to‐high heritability (ABLUP: ${\hat{h}}_{\mathrm{ind}}^{2}$ = 0.25–0.47; GBLUP: ${\hat{h}}_{\mathrm{ind}}^{2}$ = 0.20–0.29) for the cumulative number of weevil attacks (CWA), wood quality traits (Velocity_16_; Density_15_), height growth (Height_15_), and height‐to‐DBH ratio (Height_15_/DBH_15_), whereas microfibril angle (MFA_15_) was under low additive genetic control (the additive variance was not significantly different from zero). For DBH_15_, the estimated heritability was null and the type‐B correlation was zero (${\hat{r}}_{B}$ = 0) due to large genotype × environment interaction (GxE). Using the GBLUP method, weevil resistance (CWA), acoustic velocity (Velocity_16_), and average wood density (Density_15_) had the highest heritabilities (${\hat{h}}_{\mathrm{ind}}^{2}$ = 0.26–0.29). The lowest GxE estimates were also found for these traits (${\hat{r}}_{B}$ = 0.76–0.86 using GBLUP), indicating little rank changes of families between sites. GxE was moderate for Height_15_ and Height_15_/DBH_15_ (${\hat{r}}_{B}$ = 0.52–0.56 using GBLUP) and was higher for MFA_15_ and DBH_15_ (${\hat{r}}_{B}$ < 0.43 using GBLUP).

**Table 2:** Individual narrow‐sense heritability (${\hat{h}}_{\mathrm{ind}}^{2}$) and type‐B genetic correlation (${\hat{r}}_{B}$) estimates (standard errors in parentheses) using the single‐trait ABLUP and GBLUP methods for the across‐site analyses^a^. For heritability estimates, the significance of the additive variance component is shown (see Table S4). For type‐B genetic correlations, the significance of the site × additive variance component is shown^b^. A significant site × additive variance indicates significant genotype‐by‐environment interaction (i.e., smaller values of ${\hat{r}}_{B}$)

Trait^c^	ABLUP		GBLUP	
	${\hat{h}}_{\mathrm{ind}}^{2}$	${\hat{r}}_{B}$	${\hat{h}}_{\mathrm{ind}}^{2}$	${\hat{r}}_{B}$
Velocity_16_	0.37 (0.12)**	0.79 (0.15)	0.29 (0.08)***	0.76 (0.16)
Density_15_	0.25 (0.11)*	0.65 (0.2)*	0.26 (0.08)**	0.76 (0.17)
MFA_15_	0.08 (0.06)	0.47 (0.32)*	0.06 (0.05)	0.43 (0.32)**
DBH_15_	0.00 (0.00)	0.00 (0.00)***	0.00 (0.00)	0.00 (0.00)**
Height_15_	0.47 (0.16)**	0.65 (0.15)***	0.22 (0.08)**	0.52 (0.17)***
Height_15_/DBH_15_	0.40 (0.14)**	0.68 (0.16)**	0.20 (0.08)*	0.56 (0.20)**
CWA	0.47 (0.12)***	0.97 (0.08)	0.27 (0.07)***	0.86 (0.15)

a The model fitted is described in Equation (2).

b Level of statistical significance: *p < 0.05; **p < 0.01; ***p < 0.001.

c See Table 1 for full description of traits.

### 3.2 Correlations between weevil resistance, growth, and wood quality traits

Phenotypic and genetic correlations between traits using the GBLUP method are presented for each site separately, in Table 3 for GPI, the most severely affected site by weevil attacks, and in Table 4 for STM, the site that was moderately affected. Results using ABLUP were similar (GPI: Table S5; STM: Table S6), and so we present below only the results using GBLUP. Weevil resistance was significantly correlated with growth and wood quality traits. On both sites, we found a moderate negative genetic correlation (GPI: ${\hat{r}}_{a}$ = −0.52; STM: ${\hat{r}}_{a}$ = −0.57) between the cumulative number of weevil attacks (CWA) and acoustic velocity (Velocity_16_), and a strong negative genetic correlation between CWA and Height_15_/DBH_15_ (GPI: ${\hat{r}}_{a}$ = −0.99; STM: ${\hat{r}}_{a}$ = −0.79). There was a negative genetic correlation between CWA and Height_15_ on both sites (GPI: ${\hat{r}}_{a}$ = −0.69; STM: ${\hat{r}}_{a}$ = −0.60), although this genetic correlation was only significant on the most severely affected site GPI by weevil attacks. Conversely, there was a positive, but not significant, genetic correlation between CWA and DBH_15_ (GPI: ${\hat{r}}_{a}$ = 0.49; STM: ${\hat{r}}_{a}$ = 0.55). Overall, these results indicated that weevil‐resistant genotypes were taller, with larger height‐to‐DBH ratio and higher wood stiffness as measured by acoustic velocity.

**Table 3:** Site GPI: phenotypic (${\hat{r}}_{p}$, above diagonal) and genetic correlations (${\hat{r}}_{a}$, below diagonal) between traits calculated with the GBLUP method^a^. Diagonal elements indicate the single‐site narrow‐sense heritability (${\hat{h}}_{ind\;ss}^{2}$) for each trait. Standard errors of estimates are in parentheses. Genetic and phenotypic correlations were tested for significance. For ${\hat{h}}_{ind\;ss}^{2}$, the significance of the additive variance component is shown^b^

Trait^c^	Velocity_16_	Density_15_	MFA_15_	DBH_15_	Height_15_	Height_15_/DBH_15_	CWA
Velocity_16_	0.38 (0.09)***	0.32 (0.06)***	−0.10 (0.05)	−0.16 (0.06)**	0.28 (0.06)***	0.41 (0.05)***	−0.19 (0.06)*
Density_15_	0.61 (0.14)***	0.49 (0.10)***	−0.04 (0.05)	−0.46 (0.05)***	−0.07 (0.07)	0.39 (0.05)***	−0.12 (0.06)
MFA_15_	−0.16 (0.38)	−0.11 (0.40)	0.06 (0.05)	0.02 (0.05)	0.01 (0.05)	−0.03 (0.05)	0.04 (0.05)^†^
DBH_15_	−0.02 (0.28)	−0.38 (0.23)	−0.52 (0.48)	0.18 (0.09)*	0.40 (0.05)***	−0.56 (0.04)***	0.11 (0.06)
Height_15_	0.6 (0.17)**	0.00 (0.18)	0.29 (0.34)	0.33 (0.22)	0.54 (0.09)***	0.52 (0.05)***	−0.48 (0.05)***
Height_15_/DBH_15_	0.62 (0.14)***	0.36 (0.16)*	0.37 (0.34)	−0.35 (0.21)	0.74 (0.12)***	0.44 (0.09)***	−0.54 (0.04)***
CWA	−0.52 (0.18)*	−0.20 (0.19)	−0.14 (0.38)†	0.49 (0.25)	−0.69 (0.12)***	−0.99 (0.04)***	0.44 (0.09)***

a The model fitted is described in Equation (5).

b Level of statistical significance: *p < 0.05; **p < 0.01; ***p < 0.001; ^†^Convergence failed.

c See Table 1 for full description of traits.

**Table 4:** Site STM: phenotypic (${\hat{r}}_{p}$, above diagonal) and genetic correlations (${\hat{r}}_{a}$, below diagonal) between traits calculated with the GBLUP method^a^. Diagonal elements indicate the single‐site narrow‐sense heritability (${\hat{h}}_{ind\;ss}^{2}$) for each trait. Standard errors of estimates are in parentheses. Genetic and phenotypic correlations were tested for significance. For ${\hat{h}}_{ind\;ss}^{2}$, the significance of the additive variance component is shown^b^

Trait^c^	Velocity_16_	Density_15_	MFA_15_	DBH_15_	Height_15_	Height_15_/DBH_15_	CWA
Velocity_16_	0.47 (0.11)***	0.26 (0.07)***	−0.32 (0.05)***	−0.18 (0.08)*	0.23 (0.07)*	0.41 (0.07)***	−0.22 (0.07)**
Density_15_	0.16 (0.27)	0.21 (0.09)***	0.14 (0.06)	−0.43 (0.06)***	−0.16 (0.07)**	0.40 (0.06)***	−0.22 (0.06)***
MFA_15_	−0.78 (0.16)**	0.18 (0.32)	0.19 (0.08)***	−0.07(0.06)	−0.14 (0.06)	−0.01 (0.06)	−0.02 (0.06)
DBH_15_	−0.29 (0.32)	0.08 (0.38)	0.71 (0.32)*	0.14 (0.08)**	0.62 (0.04)***	−0.69 (0.04)***	0.25 (0.06)***
Height_15_	0.62 (0.22)*	0.15 (0.36)	−0.16 (0.33)	0.05 (0.44)	0.21 (0.10)**	0.11 (0.07)	−0.23 (0.06)**
Height_15_/DBH_15_	0.58 (0.19)*	0.02 (0.28)	−0.65 (0.23)*	−0.69 (0.19)*	0.71 (0.24)*	0.34 (0.10)***	−0.52 (0.05)***
CWA	−0.57 (0.21)*	−0.06 (0.30)	0.25 (0.29)	0.55 (0.27)	−0.60 (0.25)	−0.79 (0.12)***	0.29 (0.10)***

a The model fitted is described in Equation (5).

b Level of statistical significance: *p < 0.05; **p < 0.01; ***p < 0.001.

c See Table 1 for full description of traits.

Significant genetic correlations between wood quality and growth traits were found for both sites. Acoustic velocity (Velocity_16_) was strongly positively correlated with Height_15_ and Height_15_/DBH_15_ for both sites (${\hat{r}}_{a}$ > 0.58). Average wood density (Density_15_) was also weakly positively correlated with Height_15_/DBH_15_ (${\hat{r}}_{a}$ = 0.36) for site GPI, but this genetic correlation was not significant for site STM. MFA was strongly positively correlated with DBH_15_ (${\hat{r}}_{a}$ = 0.71) and negatively correlated with Height_15_/DBH_15_ (${\hat{r}}_{a}$ = −0.65) for site STM, but these correlations were not significant for site GPI.

### 3.3 Accuracy of single‐trait genomic selection models

**Figure 4:**
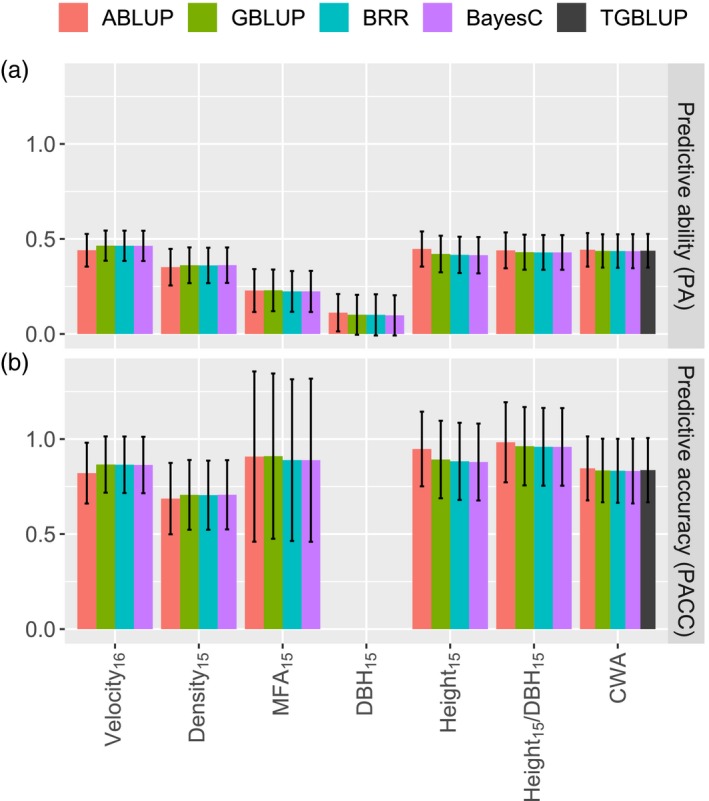
(a) Predictive ability (PA) and (b) predictive accuracy (PACC) of the single‐trait genomic selection models (GBLUP, BRR, BayesCπ) and the conventional pedigree‐based model (ABLUP) tested in this study. For the cumulative number of weevil attacks (CWA), three models accounted for ordinal data type, namely the threshold GBLUP model (TGBLUP), BRR, and BayesCπ, while ABLUP and GBLUP assumed that errors were normally distributed. Error bars indicate the standard errors of the estimates. The PACC of models for the trait DBH_15_ was not calculated because the estimated heritability was null. See Table 1 for full description of traits

The four tested single‐trait genomic selection (GS) methods (GBLUP, TGBLUP, BRR, BayesCπ) and the conventional pedigree‐based method (ABLUP) resulted in similar predictive abilities and predictive accuracies. The PA, which was defined as the correlation between the predicted breeding values for the validation trees and the phenotypic values, ranged from 0.10 for DBH_15_ to 0.46 for Velocity_16_ (Figure 4). Low heritability traits (MFA_15_, DBH_15_) had the smallest PA, while PA for all other traits was above 0.35. After standardizing PA with the square root of heritability, the estimated predictive accuracy (PACC) was obtained and it was high for all traits (PACC > 0.69). PACC was very high (0.97) for Height_15_/DBH_15_ and above 0.80 for CWA, Velocity_16_, Height_15_, and MFA_15_. However for MFA_15_, the standard error of the estimated PACC was high, which is likely due to a low heritability estimate with large standard error. PACC for DBH_15_ was not estimated because of the null heritability observed for this trait.

For the cumulative number of weevil attacks (CWA), the three methods that considered ordinal data, namely TGBLUP, BRR, and BayesCπ, had PA and PACC similar to those of GBLUP, which assumed normality of residuals (Figure 4). In addition, genomic‐estimated breeding values obtained from TGBLUP and GBLUP were highly correlated (Pearson *r* = 0.997), and the heritability estimates were within the same range (GBLUP: ${\hat{h}}_{\mathrm{ind}}^{2}$ = 0.27 (0.07); TGBLUP: ${\hat{h}}_{\mathrm{ind}}^{2}$ = 0.30 (0.08)). Thus, the assumption of the normality of residuals in GBLUP (Figure S8) did not appear to affect heritability estimates and the performance of the model in our dataset.

### 3.4 Accuracy of multi‐trait genomic selection models

**Figure 5:**
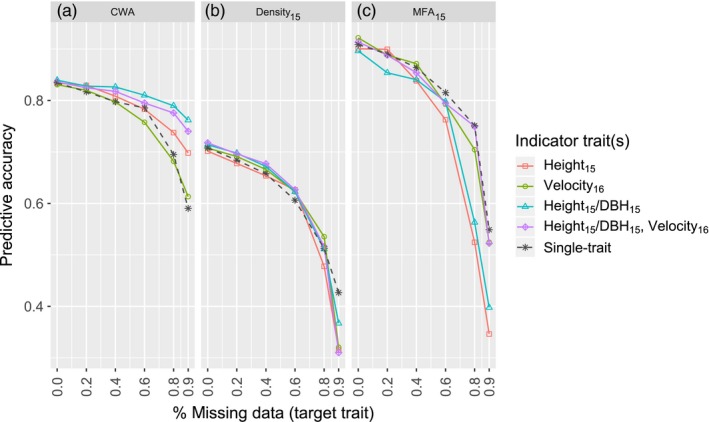
Predictive accuracy (PACC) of GBLUP multi‐trait genomic selection models for predicting the target traits: (a) the cumulative number of weevil attacks (CWA); (b) Density_15_; and (c) MFA_15_. The different colored lines represent different multi‐trait models with different indicator traits. The dashed gray line is the single‐trait GBLUP model. The percentage of missing phenotypic data for the target trait in the training sets was varied from 0% to 90% (*x*‐axis), while 100% of the training data was retained for the indicator traits. See Table 1 for full description of traits

Multi‐trait GBLUP models were used to predict the breeding values of a target trait (CWA, Density_15_, or MFA_15_), when combined with genetically correlated indicator traits (Height_15_, Velocity_16_, Height_15_/DBH_15_ ratio). The cumulative number of weevil attacks (CWA), average wood density at age 15 (Density_15_), and microfibril angle at age 15 (MFA_15_) were chosen as target traits because they are rather cumbersome and expensive to assess on a large number of trees, whereas indicator traits are easier to track for the majority of trees in a breeding population. The multi‐trait models were compared with the single‐trait GBLUP models. As expected, when the percentage of missing phenotypic data increased from 0% to 90% for the target traits CWA, Dens_15_, and MFA_15_, the predictive accuracy (PACC) of the single‐trait models (dashed gray line in Figure 5) sharply dropped from 0.83 to 0.59, from 0.71 to 0.43, and from 0.91 to 0.55, respectively. Similar trends were found for the PA (Figure S9).

The PACC of multi‐trait models was not improved over single‐trait models when all of the phenotypic data for the target trait were included for model training (0% missing data) (Figure 5; standard errors are given in Table S7). However, for the target trait CWA with 40% or more missing data, the accuracy of multi‐trait models was improved as compared with that of the single‐trait model (Figure 5a). PACC was the highest when CWA was coupled with the highly genetically correlated Height_15_/DBH_15_ ratio ($\bar{{\hat{r}}_{a}}$ = −0.89, average of single‐site estimates) as an indicator trait (blue line in Figure 5a). For this two‐trait model, PACC was maintained high (0.74) and decreased only marginally when 90% of CWA data was missing, as compared with the full dataset. The two‐trait model with Height_15_ as an indicator trait ($\bar{{\hat{r}}_{a}}$ = −0.65, red line) outperformed the single‐trait model only when CWA had 80% or more missing data. When CWA was coupled with the moderately correlated trait Velocity_16_ ($\bar{{\hat{r}}_{a}}$ = −0.55, green line), PACC was similar to that of the single‐trait model. The three‐trait model including traits CWA, the Height_15_/DBH_15_ ratio, and Velocity_16_ (purple line) outperformed the single‐trait model for 40% or more missing data, but its PACC was slightly lower than that of the two‐trait model considering CWA and the Height_15_/DBH_15_ ratio (blue line).

For the target traits Density_15_ and MFA_15_ (Figure 5b,c), multi‐trait models did not improve PACC over single‐trait GBLUP, even when the target trait had large amount of missing data. For MFA_15_, some of the multi‐trait models showed even a lower PACC than the single‐trait models.

### 3.5 Genetic gains and multi‐trait selection indices

The expected genetic gains, expressed as a percentage of the phenotypic mean when selecting the top 5% trees for each trait separately (single‐trait selection), are given in Table S8. For the cumulative number of weevil attacks (CWA), a positive gain represents a reduction of the number of attacks (i.e., higher resistance). CWA could be improved by as much as 78% (ABLUP) or 66% (GBLUP), while other traits showed much smaller gains in the 4%–20% range. The large observed gains for CWA are likely due to a moderate‐to‐high heritability of CWA, a large coefficient of phenotypic variation (Table 1), and the non‐normal distribution of the trait.

The expected gains from the multi‐trait selection indices (SIs) are shown in Table 5. Results from ABLUP and GBLUP were similar, and so, we present only the GBLUP results below. The first selection index scenario (SI‐1) presents the planned breeding focus and put most emphasis on weevil resistance (*w*_2_ = 0.6), followed by Height_15_ (*w*_1_ = 0.3), and by Velocity_16_ (*w*_3_ = 0.1). This scenario achieved the largest gains in terms of reduction of cumulative weevil attacks (CWAs) (83% of maximum possible gains from single‐trait selection in Table S8), while still yielding desirable gains for Height_15_ (86% of maximum) and Velocity_16_ (74% of maximum). The SI‐2 scenario that simultaneously maximized the genetic gain in Height_15_, Velocity_16_, and CWA reached as much as 89%, 87%, and 76% of the maximum possible gains for each trait, respectively. In this scenario, MFA_15_ was also improved by 5.6% (62% of maximum). For SI‐1 and SI‐2, the gain in Density_15_ was marginal. For SI‐3, Density_15_ was added as an important trait to maximize along with Height_15_, Velocity_16_, and CWA. This SI scenario slightly improved gains in Density_15_ by 2.6% (51% of maximum), but at the cost of smaller improvements for Height_15_, Velocity_16_, CWA, and MFA_15_. In all three SIs, stem taper, as estimated by the Height_15_/DBH_15_ ratio, was improved (i.e., lower stem taper) by ~10%.

**Table 5:** Genetic gains for each trait^a^ when selecting the top 5% trees in three selection index scenarios (SIs) using the ABLUP and GBLUP methods. Gains are expressed as a percentage of the phenotypic mean. A positive percentage indicates an improvement in the value of the trait. DBH_15_ was not considered because of the null heritability and associated null genetic gains

Selection index^b^	Velocity_16_ (%)	Density_15_ (%)	MFA_15_ c (%)	Height_15_ (%)	Height_15_/DBH_15_ (%)	CWA^c^ (%)
ABLUP						
SI−1: emphasis on weevil resistance (*w*_1_ = 0.3; *w*_2_ = 0.6; *w*_3_ = 0.1; *w*_4_ = 0)	4.27	0.08	1.70	12.13	10.97	67.97
SI−2: maximize Height_15_, CWA, Velocity_16_ (*w*_1_ = 0.25;* w*_2_ = 0.4;* w*_3_ = 0.35;* w*_4_ = 0)	6.32	0.14	4.91	12.36	11.09	56.89
SI−3: maximize Height_15_, CWA, Velocity_16_, Density_15_ (*w*_1_ = 0.2;* w*_2_ = 0.3;* w*_3_ = 0.25;* w*_4_ = 0.25)	5.77	1.87	0.25	11.51	11.10	53.77
GBLUP						
SI−1: emphasis on weevil resistance (*w*_1_ = 0.3;* w*_2_ = 0.6;* w*_3_ = 0.1;* w*_4_ = 0)	5.30	0.62	3.61	7.82	9.74	54.57
SI−2: maximize Height_15_, CWA, Velocity_16_ (*w*_1_ = 0.3;* w*_2_ = 0.35;* w*_3_ = 0.35;* w*_4_ = 0)	6.17	0.51	5.62	8.10	10.30	50.33
SI−3: maximize Height_15_, CWA, Velocity_16_, Density_15_ (*w*_1_ = 0.25;* w*_2_ = 0.25;* w*_3_ = 0.25; * w*_4_ = 0.25)	5.60	2.60	2.09	7.93	10.18	45.84

a See Table 1 for full descriptions of traits.

b Index selection formula (Equation 10): $\mathrm{SI}=w_{1}{\mathrm{Height}}_{15\mathrm{EBV}}-w_{2}{\mathrm{CWA}}_{6.15\mathrm{EBV}}+w_{3}{\mathrm{Velocity}}_{16\mathrm{EBV}}+w_{4}{\mathrm{Density}}_{15\mathrm{EBV}},$ where ${\mathrm{Height}}_{15\mathrm{EBV}}$,${\mathrm{CWA}}_{6.15\mathrm{EBV}}$, ${\mathrm{Velocity}}_{16\mathrm{EBV}}$, and ${\mathrm{Density}}_{15\mathrm{EBV}}$ are the BLUP estimated breeding values from the single‐trait ABLUP (EBVs) or GBLUP (GEBVs) analysis for the corresponding trait (Equation 2).

c For MFA and CWA, an improvement (positive percentage) is associated with a decreasing value of the trait (i.e., a reduction of the microfibril angle and a reduction of the cumulative number of weevil attacks, respectively).

## 4 DISCUSSION

### 4.1 Genetic control of weevil resistance and its relationships to growth and wood quality traits

Tree breeding uses the existing natural intraspecific variation to identify superior genotypes with desirable attributes. In this context, the heritability of natural resistance to pests may be seen as an indicator for the evolutionary potential of the species (Charmantier & Garant, 2005; Geber & Griffen, 2003). In a recently introduced species such as Norway spruce to North America, natural selection for resistance to native pests may have acted only for one or two generations at best. Nevertheless, we showed that resistance to white pine weevil attack was under moderate‐to‐high genetic control. Individual heritability estimates (ABLUP) were slightly higher than in an earlier study by Mottet et al. (2015), who combined data from more families and tests than this study, but values were in the same range than those observed in the native interior spruce (King et al., 1997) and slightly higher than those in the native Sitka spruce (King, 2004). In its native range, Norway spruce suffers damages from another weevil species, the large pine weevil (*Hylobius abietis*), mostly at the seedling stage after clearfelling operations (Day & Leather, 1997). Although both weevil species do not attack trees at the same developmental stage, both feed on the bark and phloem, so it is likely that resistance mechanisms to different weevils are partly related. Zas et al. (2017) found moderate family heritability and low GxE for resistance to the pine weevil in Norway spruce. Norway spruce has also faced outbreaks of bark beetles, such as *Ips imitinus* and *Ips typographus*, after major storms such as those that occurred in Central Europe in the 1990s (Wermelinger, 2004). Resin canal traits relevant for constitutive resistance against bark beetles were found to be under strong genetic control (Rosner & Hannrup, 2004). Thus, it is likely that genetic variation at resistance genes for the North American white pine weevil was already available at the time of introduction of Norway spruce in North America, as the result of natural selection against insect pests in Europe, with opportunities for rapid change in allele frequencies at resistance loci.

In our study, both CWA and tree height at age 15 were under significant genetic control and the genetic correlation between these traits was negative, but significant only for the site that suffered the most frequent weevil attacks (GPI). These results are not surprising given that height growth is mechanistically stunted in consequence of attack, while the tree continues to grow in DBH and volume. Indeed, with increasing age, a higher height/DBH ratio and thus a lower stem taper was observed in more weevil‐resistant trees (Holst, 1955; Mottet et al., 2015; this study). King et al. (1997) also reported negative genetic correlations between CWA and tree height for interior spruce in British Columbia, both before and after the occurrence of weevil attacks. They concluded that inherently faster growing families have higher level of genetic resistance. In Norway spruce, Mottet et al. (2015) found negligible genetic correlations between weevil attacks and tree height measured before the majority of weevil attacks (age 5) and concluded that genetic improvement for resistance to white pine weevil would not adversely affect growth. On the other hand, the strong positive genetic correlation between resistance to weevil attacks and height at age 15 found on site GPI in this study may indicate that they are controlled by common genes, which was also suggested by an earlier QTL study in interior spruce (Porth et al., 2012). In addition, resistance mechanisms to European bark beetles such as resin canal traits were found to be positively genetically correlated with both height growth and DBH (Rosner & Hannrup, 2004). Overall, our results suggest that accelerated breeding of resistant seed stock through genomic selection tools will result in taller trees, either because the leaders of resistant trees will be less affected by attacks, or because alleles underlying growth genes will be simultaneously favored.

The genetic control of variation in wood quality traits, such as average wood density and acoustic velocity as a proxy for wood stiffness, was in the same range as that for weevil resistance. Compared to recent wood quality studies of Norway spruce from Scandinavia (Chen et al., 2014, 2015), our heritability estimates were lower for average wood density, but higher for acoustic velocity. Heritability estimates for MFA were low compared with those reported in previous studies (Chen et al., 2014; Lenz et al., 2010), which is most likely related to the measuring approach used in the present study where only the last ring was assessed. The moderate heritability and the resulting sizeable genetic gain observed here for acoustic velocity make it a promising quick‐assessment trait for improving wood stiffness and product quality (Lenz et al., 2013). In addition, wood stiffness can be improved together with weevil resistance since acoustic velocity was negatively genetically correlated with the cumulative number of weevil attacks. Moreover, weevil resistance and wood traits showed low genotype‐by‐environment interactions, which should facilitate reforesting selected planting stock across larger breeding zones. Average wood density did not appear to be the best trait for overall improvement of Norway spruce in the tested conditions given its negative correlations with growth traits and slightly higher genotype‐by‐environment interaction. Overall, given their positive genetic correlations and moderate‐to‐high heritability, weevil resistance, acoustic velocity, and height growth appear as excellent candidate traits for simultaneous genetic improvement.

### 4.2 Genomic selection models for accurate and hastened selection of best genotypes

Genomic selection can significantly shorten breeding cycles through the prediction of breeding values of nonphenotyped material using their genomic profiles and allows screening more candidates for increase selection intensity or multi‐trait selection. Compared with the conventional pedigree‐based approach (ABLUP), genomic selection models using GBLUP, BRR, or BayesCπ had comparable PA and accuracy, confirming early proof‐of‐concept studies in other conifers and spruces (Beaulieu, Doerksen, Clément, et al., 2014; Beaulieu, Doerksen, MacKay, et al., 2014; Gamal El‐Dien et al., 2015; Lenz et al., 2017; Ratcliffe et al., 2015). In BRR and BayesCπ, we did not fit genotype‐by‐environment interactions as opposed to ABLUP and GBLUP methods, but this did not affect PA nor predictive accuracy.

Predictive ability was related to heritability and was lowest for the low heritability traits MFA_15_ and DBH_15_. This is because the proportion of phenotypic variation that can be explained by additive genetic effects is smaller for these traits. Comparisons of predictive accuracy of breeding values across studies are difficult because of the absence of a standard way of determination. For similar growth and wood quality traits, predictive accuracy was slightly higher than previously observed for native Norway spruce (Chen et al., 2018) or for white spruce (Beaulieu, Doerksen, Clément, et al., 2014; Beaulieu, Doerksen, MacKay, et al., 2014; Gamal El‐Dien et al., 2015; Ratcliffe et al., 2015), and it was lower and more variable than that observed for black spruce (*Picea mariana* [Mill.] B.S.P.) (Lenz et al., 2017). In contrast to those previous GS studies, we assumed herein that true breeding values were unknown and calculated the predictive accuracy by dividing the PA by the square root of heritability (Dekkers, 2007; Legarra et al., 2008). Hence, predictive accuracy should not be correlated with heritability, but depends on other characteristics of the dataset, such as the accuracy of phenotypic measurements, the effective population size, and the genetic architecture of traits (Grattapaglia & Resende, 2011). However, the precision of the present estimates of predictive accuracy may be affected by the precision of heritability estimates.

In theory, GS should perform better than pedigree‐based models because it allows correcting pedigree errors and capturing the within‐family variation resulting from Mendelian segregation (Grattapaglia et al., 2018). Thus, in a forward selection scenario with nonphenotyped young material, the selection of the best individuals within families becomes possible with GS. In the present context of small size of the breeding population, we conclude that the estimated genomic predictions for resistance to weevil attack, tree height, height/DBH ratio, and wood quality traits allow for accurate and hastened selection of best candidates based on their genomic profiles.

### 4.3 Evidence for polygenic control of weevil resistance in spruces

To our knowledge, the present study is the first one applying genomic selection to breed for insect resistance in conifers. We therefore had particular interest in testing different algorithms that considered the ordinal distribution of this trait and that reflected different distributions of marker effects. First, we found no advantages of using methods that accounted for ordinal data (threshold GBLUP, BRR, and BayesCπ), as compared with approaches that assumed normality of residuals (ABLUPs and GBLUPs). Second, the BayesCπ algorithm, which assumed that only a portion of genes had an effect, did not lead to improvement of PA or accuracy compared with methods assuming that all genes had small effects (GBLUPs, BRR). In BayesCπ, the estimated proportion of markers having an effect (π≅0.50) for weevil resistance was in the same range as that for other traits (Table S9). In contrast, Resende, Munoz, et al. (2012) and Resende, Resende, et al. (2012) obtained a higher predictive accuracy using BayesCπ for fusiform rust resistance in loblolly pine, which suggested the presence of large effect genes. Our findings can be explained by two possible phenomena: (a) weevil resistance is effectively controlled by many genes of small effects, with no detectable major gene effects; or (b) none of the sampled SNPs was in close linkage with genes having effects for resistance. It is difficult to discriminate between both hypotheses, especially since GS models with the current genome coverage and small size of the breeding population should mostly retrace relatedness between trees from long‐range linkage disequilibrium (e.g., Beaulieu, Doerksen, MacKay, et al., 2014; Lenz et al., 2017) and could thus provide high predictive accuracies following both sets of conditions. However, polygenic control of weevil resistance seems plausible since earlier reports associated resistance to weevil attack with different constitutive and induced mechanisms. Physical defense barriers to weevil were described through sclereids and stone cells in the bark of sitka and hybrid spruces (King, Alfaro, Lopez, & Van Akker, 2011; Whitehill et al., 2016). In addition, chemical defense strategies are also at play through the presence of abundant constitutive resin ducts (King et al., 2011; Rosner & Hannrup, 2004) and the additional formation of traumatic resin ducts (Poulin, Lavallee, Mauffette, & Rioux, 2006), together with increase in terpenoid metabolite production (Robert et al., 2010) following insect feeding. Porth et al. (2012) identified many candidate genes for weevil resistance in interior spruce, including some master regulatory genes. Moreover, transcriptome studies in Sitka spruce revealed several thousand differentially expressed genes between resistant and sensible genotypes, as well as following weevil wounding and feeding (Ralph et al., 2006; Whitehill et al., 2019). Hence, weevil resistance in Norway spruce is most likely polygenic given the complex nature of physical and chemical defense mechanisms, but whether some larger gene effects exist remains to be elucidated.

### 4.4 Multi‐trait GS as a tool to improve accuracy of scarcely phenotyped traits

The joint modeling of multiple traits can benefit from genetic correlations between traits and increase predictive accuracy (Calus & Veerkamp, 2011; Guo et al., 2014; Jia & Jannink, 2012). In *Eucalyptus*, Cappa et al. (2018) reported modest improvements in the predictive accuracy of breeding values from multi‐trait over single‐trait GS models (~2%–4%) when a low heritability target trait (tree height) was coupled with a highly genetically correlated trait (DBH, *r_a_* = 0.92). Other empirical plant or tree breeding studies found little benefits of using multi‐trait GS to predict nonphenotyped selection candidates when 100% of the individuals in the training set were phenotyped (Bao et al., 2015; Cheng et al., 2018; Fernandes et al., 2018; Jia & Jannink, 2012; Schulthess et al., 2016). Similarly, we found that multi‐trait GBLUP did not outperform single‐trait models when all individuals in the training set were phenotyped for the target trait. Thus, in cases when phenotypic information is balanced across traits, single‐trait modeling is the recommended method given that they harbor reduced model complexity (Schulthess et al., 2016).

Multi‐trait GS models present an interesting strategy to cope with traits that have large amount of missing values because they are difficult or costly to measure, such as traits related to resistance to biotic and abiotic factors, or wood quality traits. We found that when we included a highly genetically correlated growth trait (Height_15_/DBH_15_ ratio or Height_15_) as an indicator trait, the multi‐trait models predicted resistance to weevil attacks more accurately when there was a large amount of missing data for this trait. However, the advantage of multi‐trait over single‐trait GS disappeared as the genetic correlation between weevil resistance and the indicator trait decreased to $\bar{{\hat{r}}_{a}}$ = −0.55 when using Velocity_16_ as the indicator trait. Similarly, there was no advantage of using multi‐trait models to predict the target traits Density_15_ or MFA_15_, which was likely due to weak or inconsistent genetic correlations across sites between the target and indicator traits. Previous studies similarly found that multi‐trait models performed best when the genetic correlation between traits was high (*r_a_* > 0.5; Calus & Veerkamp, 2011; Guo et al., 2014; Montesinos‐López et al., 2016). Thus, our results and those of previous studies suggest that, in the case of a target trait with moderate heritability such as weevil resistance, multi‐trait GS models are only advantageous when a highly genetically correlated indicator trait is available.

In a breeding context, our results open up possibilities to consider material tested on sites where no record of weevil attacks have been taken. Accurate phenotyping of weevil resistance requires numerous visits at each trial and at different plantation ages. Furthermore, an appropriate level of attack at each site, ideally more than 50% of attacked trees, is desirable to properly evaluate genetic resistance. These constraints imply that resistance to weevil attacks is often recorded only for a part of available test sites and material. In such cases, multi‐trait GS models can be used to more accurately predict weevil resistance for the trees in nonphenotyped sites when an indicator trait has been measured in all sites (Montesinos‐López et al., 2016). Furthermore, the low genotype‐by‐environment interaction of resistance to weevil attacks found in this study and in Mottet et al. (2015) would ensure relatively high accuracies of predictions between sites (Beaulieu, Doerksen, MacKay, et al., 2014; Lenz et al., 2017).

Due to convergence issues for the three‐trait models, we did not account for genotype‐by‐environment interactions (GxE) in our multi‐trait models. We adjusted the phenotypes for site and block effects, but this standardization does not control for GxE due to rank changes between sites. Nevertheless, this seemed to be a reasonable model simplification in the present case, given that we found the same predictive accuracy for single‐trait GBLUP models that accounted for GxE (Equation 2) compared with GBLUP models that used the adjusted phenotype as a response variable and did not account for GxE (Equation 11 in Appendix S2, Table S10).

Models with more than two traits have only been tested in a handful of studies (Bao et al., 2015; Schulthess et al., 2016; Tsuruta, Misztal, Aguilar, & Lawlor, 2011). In particular, Schulthess et al. (2016) did not find any benefit of using three‐trait over two‐trait models when the aim was to predict only one target trait. However, they found that the three‐trait model was better to predict two scarcely phenotyped target traits, when a third correlated trait was fully available. In this study, the three‐trait model with indicator traits Height_15_/DBH_15_ ratio and Velocity_16_ did not outperform our best two‐trait model using only Height_15_/DBH_15_ ratio as an indicator trait to predict resistance to weevil attacks. This is because Velocity_16_ was much less correlated with weevil resistance ($\bar{{\hat{r}}_{a}}$ = −0.55) than was the Height_15_/DBH_15_ ratio ($\bar{{\hat{r}}_{a}}$ = −0.89), and thus, Velocity_16_ added little information to the predictions. Because of the rapidly increasing complexity of models as the number of correlated traits increases, computer resource limitations and convergence problems due to collinearity can arise (Schulthess et al., 2016). In this study, convergence problems precluded us to fit simultaneously more than three traits. Multi‐trait Bayesian models are expected to be more efficient than multi‐trait GBLUP when the number of traits increases (Calus & Veerkamp, 2011). Overall, the benefits of adding more traits seems limited, but this needs to be further tested with different GS models and with traits combinations of different levels of heritability and genetic correlations.

### 4.5 Index selection provided positive gains for most focus traits

Index selection is a commonly used tool in tree improvement to select individuals that combine superior genetic value for several traits of interest to the breeder. This strategy usually results in lower genetic gain for each trait compared with single‐trait selection, because correlations among traits are not perfect and are often negative. Our scenario SI‐1 reflected the priorities of the Norway spruce breeders of the province of Québec. Besides the current focus on weevil resistance and growth, wood quality traits will be included in the next generation selection criteria for this plantation‐grown species to avoid a reduction in mechanical properties of lumber extracted from faster growing trees. However, determining economic weights for each trait is challenging. Economic studies have been conducted for different conifer breeding programs (e.g., Aubry, Adams, & Fahey, 1998; Ivković, Wu, McRae, & Powell, 2006; Petrinovic, Gélinas, & Beaulieu, 2009), but the resulting economic weights are not transferable to other species and regions since they depend much on the wood production and transformation systems. Because of these difficulties, economic weights have not been established yet for Norway spruce in the Canadian breeding and forestry contexts, and the relative weights that we have chosen for the SI‐1 scenario are currently the most likely to be implemented in the short term but are still of an indicative nature.

In this study, favorable correlations between weevil resistance, height growth, and acoustic velocity resulted in only minor trade‐offs among those traits in the tested selection indices. However, the improvement for DBH or the closely related volume (not estimated in this study) appears more challenging. The low genetic control and the important genotype‐by‐environment interaction observed for DBH (see also Mottet et al., 2015) make it difficult to predict the effect of selecting for weevil resistance on this trait. However, the possible loss in lumber volume would likely be compensated by less log defects due to increased resistance of plantations to weevil attacks (Daoust & Mottet, 2006). Thus, improving for weevil resistance would undoubtedly benefit Norway spruce as an exotic plantation species in eastern Canada.

## 5 CONCLUSIONS

In this study, we investigated the genetic control of weevil resistance and its relationship with wood and growth traits in Norway spruce as an exotic plantation species. We further integrated these traits into a multi‐trait genomic selection (GS) framework. We found that in such a realistic context, it was possible to improve significantly for weevil resistance using GS, given that weevil resistance was moderately to highly heritable and that it was positively genetically correlated with the height/DBH ratio and wood stiffness (acoustic velocity). By taking advantage of these existing genetic relationships, we showed that multi‐trait genomic selection models could improve the accuracy of the prediction for a scarcely phenotyped target trait (weevil resistance) by using the information from a readily available indicator trait. Finally, by combining multiple correlated traits into a selection index, we obtained the best compromise for all traits of interest that corresponded to the priorities of the breeders. Thus, this integrated approach showed how genomic selection can be used to breed simultaneously for taller, stiffer, and more weevil‐resistant Norway spruces. We conclude that single/multi‐trait GS models and index selection are efficient selection tools that can be integrated into operational breeding programs to accelerate the realization of genetic gains for most traits of interest.

Another advantage of integrating genomic selection to a breeding program is that, once the breeding population has been genotyped, models can easily be recalibrated for additional traits, such as pest resistance or resilience to episodic climate extremes such as droughts. Such traits may be costly and difficult to measure in forest trees (e.g., Housset et al., 2018) and the use of correlated indicator traits in multi‐trait models would reduce phenotyping costs. Mass phenotyping using remote sensing technologies could also help identify indicator traits that are correlated with economically important ones (Dungey et al., 2018). The increase of information from multiple correlated traits will provide opportunities to test novel multi‐trait models and to improve genomic selection accuracies. Finally, given that large numbers of candidate trees can be genotyped at the very juvenile stage, multi‐trait GS will result in highly significant reductions in testing time for such long‐lived species, while allowing to increase selection intensity compared to more conventional selection approaches.

## DATA ARCHIVING STATEMENT

SNP data and the description of the genotyping array were deposited in the European Variation Archive (EVA, https://www.ebi.ac.uk/eva/, accession number PRJEB27427). More details can be found in Azaiez et al. (2018). The phenotypic and genotypic original data belong to the Norway spruce breeding program of the province of Québec and have been stored in our institutions’ databases. It can be shared upon request to the corresponding author according to the intellectual property policies (IPP) of participating governmental institutions. Therefore, data have therefore not been deposited directly into a public domain.
