# Supplementary material for: Multi‐trait genomic selection for weevil resistance, growth, and wood quality in Norway spruce
Source: Evol Appl. 2019 Jun 20;13(1):76–94. doi: 10.1111/eva.12823 (PMC6935592; doi:10.1111/eva.12823)
Supplement: Supplementary file 1 [file EVA-13-76-s001.docx]

| **Mother/Father** | P-1 | P-2 | P-3 | P-4 | P-5 | P-6 | P-7 | P-8 | P-9 | P-10 | P-11 | P-12 | P-13 | P-29 | P-30 | P-31 | P-32 | P-33 | P-34 | P-35 |
| --- | --- | --- | --- | --- | --- | --- | --- | --- | --- | --- | --- | --- | --- | --- | --- | --- | --- | --- | --- | --- |
| P-1 |  | 17 |  |  |  |  |  |  | 20 |  |  |  |  | 14 | 20 |  |  |  |  |  |
| P-2 |  |  |  |  |  |  |  |  |  |  |  |  |  |  | 18 |  |  |  |  |  |
| P-3 |  |  |  |  |  |  |  | 17 |  |  |  |  |  |  |  |  |  |  |  |  |
| P-4 |  |  |  |  |  |  | 18 |  |  |  |  |  |  |  |  |  |  |  |  |  |
| P-5 |  |  |  |  |  |  |  |  |  |  |  |  |  |  |  |  | 20 |  |  |  |
| P-6 |  |  |  |  |  |  |  |  |  |  |  |  |  |  |  |  |  | 20 |  |  |
| P-7 |  |  |  | 16 |  |  |  |  |  |  |  |  |  |  |  |  |  |  |  |  |
| P-8 |  |  |  |  |  |  |  |  |  |  |  |  | 20 |  |  |  |  |  |  |  |
| P-9 |  |  |  |  | 16 |  |  |  |  |  |  |  |  |  |  |  |  |  |  |  |
| P-10 |  |  |  |  |  |  |  | 18 |  |  |  |  |  |  |  |  |  |  |  |  |
| P-11 |  |  |  |  |  | 19 |  |  |  | 18 |  |  |  |  |  |  |  |  |  |  |
| P-12 |  |  |  |  |  |  |  |  | 17 | 20 |  |  |  |  |  |  |  |  |  |  |
| P-13 |  |  |  |  |  |  | 19 |  | 18 |  |  |  |  |  |  |  |  |  |  |  |
| P-14 | 18 |  |  |  |  |  |  |  |  |  |  |  |  | 17 |  |  |  |  |  |  |
| P-15 |  |  |  |  |  |  | 18 |  |  |  |  |  |  | 18 |  |  |  |  |  |  |
| P-16 |  |  | 15 |  |  |  |  |  |  |  |  |  |  |  |  |  |  |  |  |  |
| P-17 |  |  |  |  |  |  |  |  |  |  |  |  |  |  |  | 17 |  |  |  |  |
| P-18 |  |  |  |  |  |  |  | 17 |  |  |  |  |  |  |  |  |  |  |  |  |
| P-19 |  |  |  |  |  |  |  |  |  |  |  |  | 17 |  |  |  | 19 |  |  |  |
| P-20 |  |  |  |  |  |  |  |  |  |  |  |  |  |  |  |  |  | 19 | 19 |  |
| P-21 |  |  |  |  |  |  |  |  | 18 | 17 |  |  |  |  |  |  |  |  |  |  |
| P-22 |  |  |  |  |  |  |  |  |  |  |  |  |  |  |  |  |  |  |  | 20 |
| P-23 |  |  |  |  |  |  |  |  |  |  | 15 |  |  |  |  |  |  |  |  |  |
| P-24 |  |  |  |  |  |  |  |  |  |  | 18 |  |  |  |  |  |  |  |  |  |
| P-25 |  |  |  |  |  |  |  |  |  |  | 20 |  |  |  |  |  |  |  |  |  |
| P-26 |  |  |  |  |  |  |  |  |  |  | 16 |  |  |  |  |  |  |  |  |  |
| P-27 |  |  |  |  |  |  |  |  |  |  | 18 | 19 |  |  |  |  |  |  |  |  |
| P-28 |  |  |  |  |  |  |  |  |  |  | 14 |  |  |  |  |  |  |  |  |  |

**Figure S1.** Partial diallel mating design. Mothers and fathers are listed in rows and columns, respectively. The gray square represent parents that were both crossed as mother and father (listed in the same order in rows and columns). The number in cells indicate the number of progeny for each cross used in data analyses. Orange and green cells indicate sensible and resistant families, respectively.


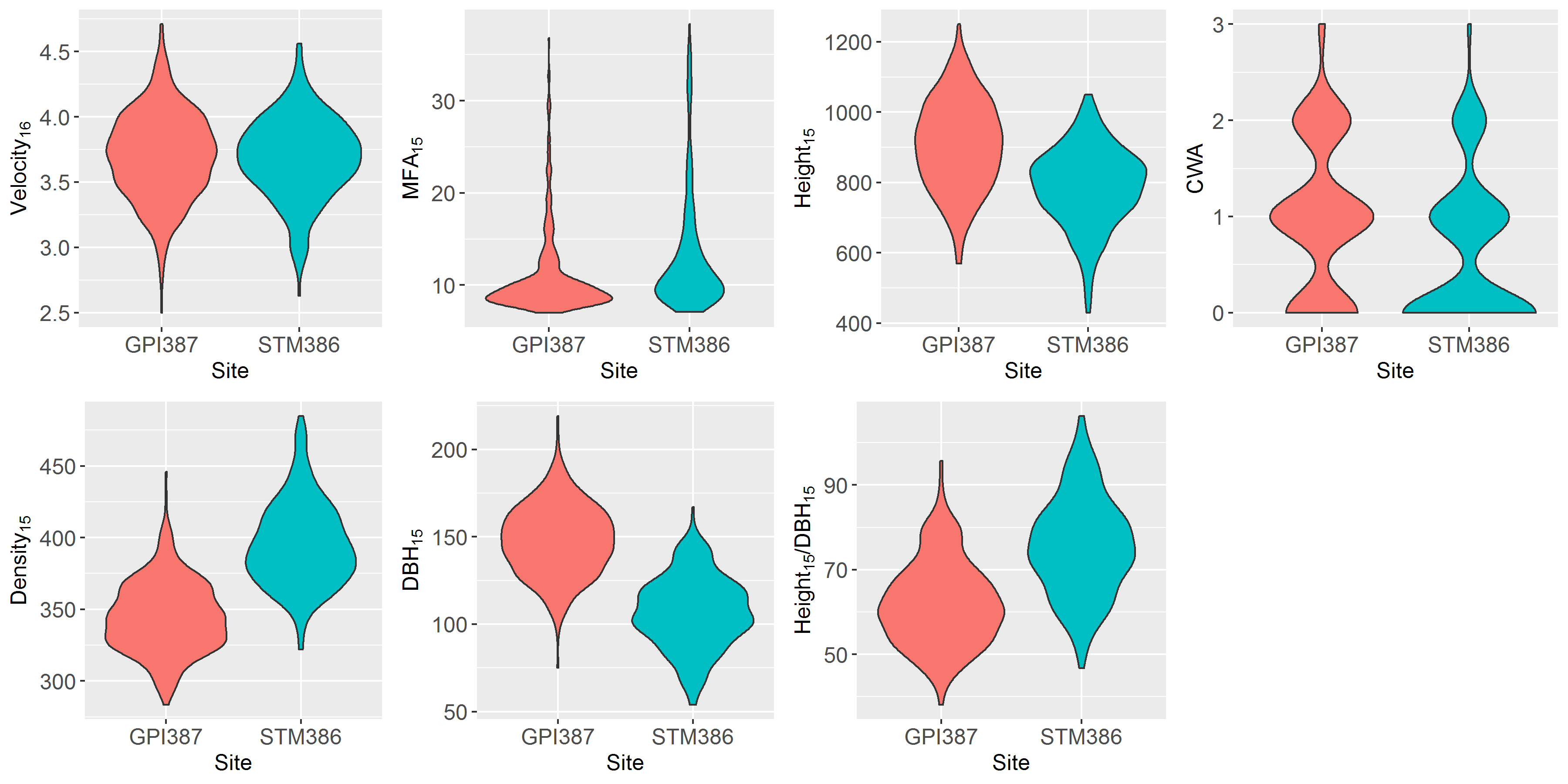


**Figure S2.** Violin plots grouped by sites GPI and STM for the traits assessed in this study. See Table 1 in the manuscript for full description of traits.

| **(A)** | **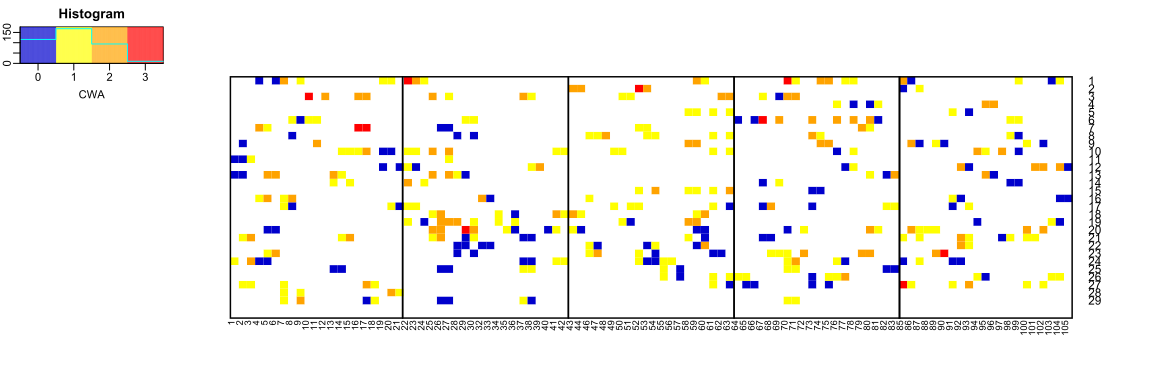** |
| --- | --- |
| **(B)** | **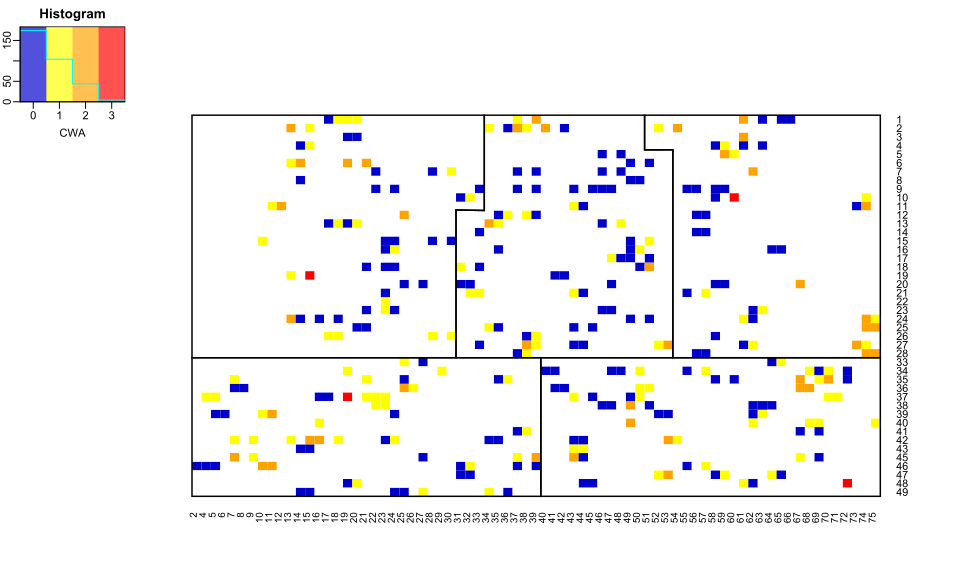** |

**Figure S3.** Spatial distribution of CWA in sites (A) GPI and (B) STM. The squares represent the blocking structure.


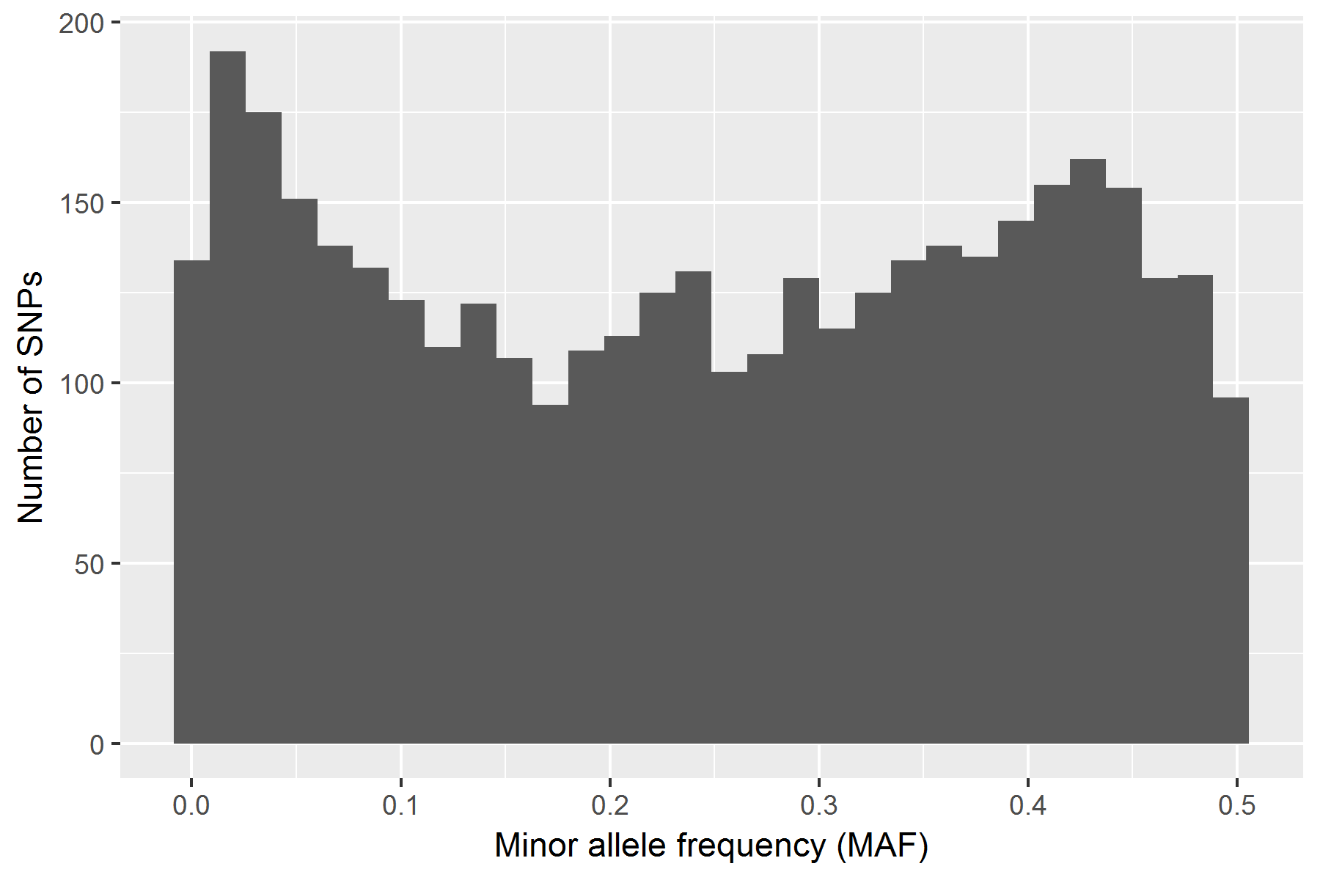


**Figure S4.** Histogram of minor allele frequency for the 3914 retained SNPs.

**
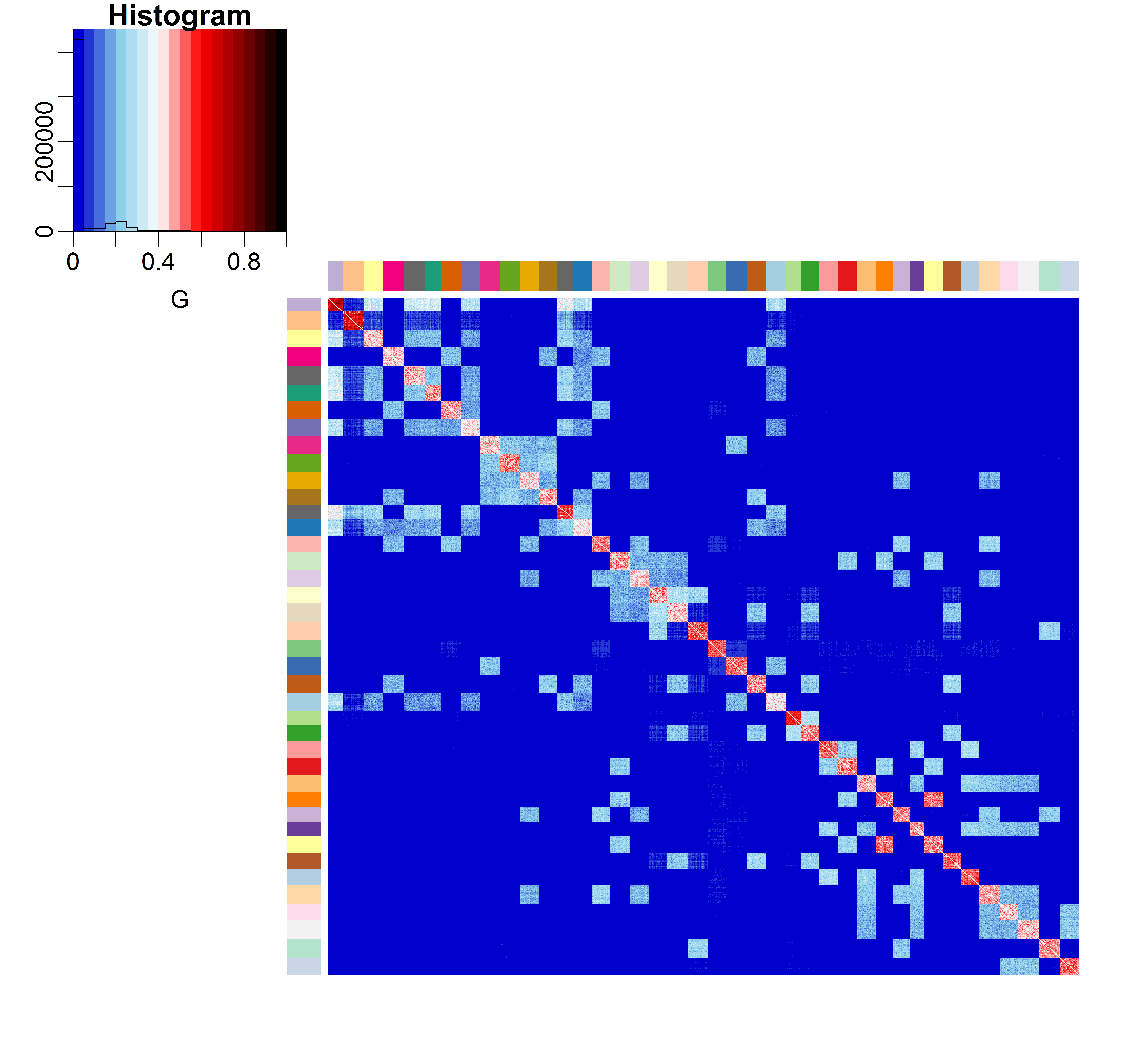
Figure S5**. Heatmap of the realized genomic relationship matrix ($\boldsymbol{G}$). The color key (top left corner) is represented as a histogram of pairwise G values. The row and column side colors represent family membership. The first 20 families are the weevil resistant families and the last 20 families are the weevil sensitive families. The 40 full-sib families appear as blocks of related individuals near the diagonal (values of $\boldsymbol{G}$ near 0.5) and families that share one parent appear as blocks of related individuals further away from the diagonal (values of $\boldsymbol{G}$ near 0.25). The mean pairwise G is 0.0304 and 0.0290 among sensitive and resistant families, respectively. The blocks of full-sib related individuals off the diagonal represent two full-sib families (Fam_30 and Fam_33) that share the same parents.


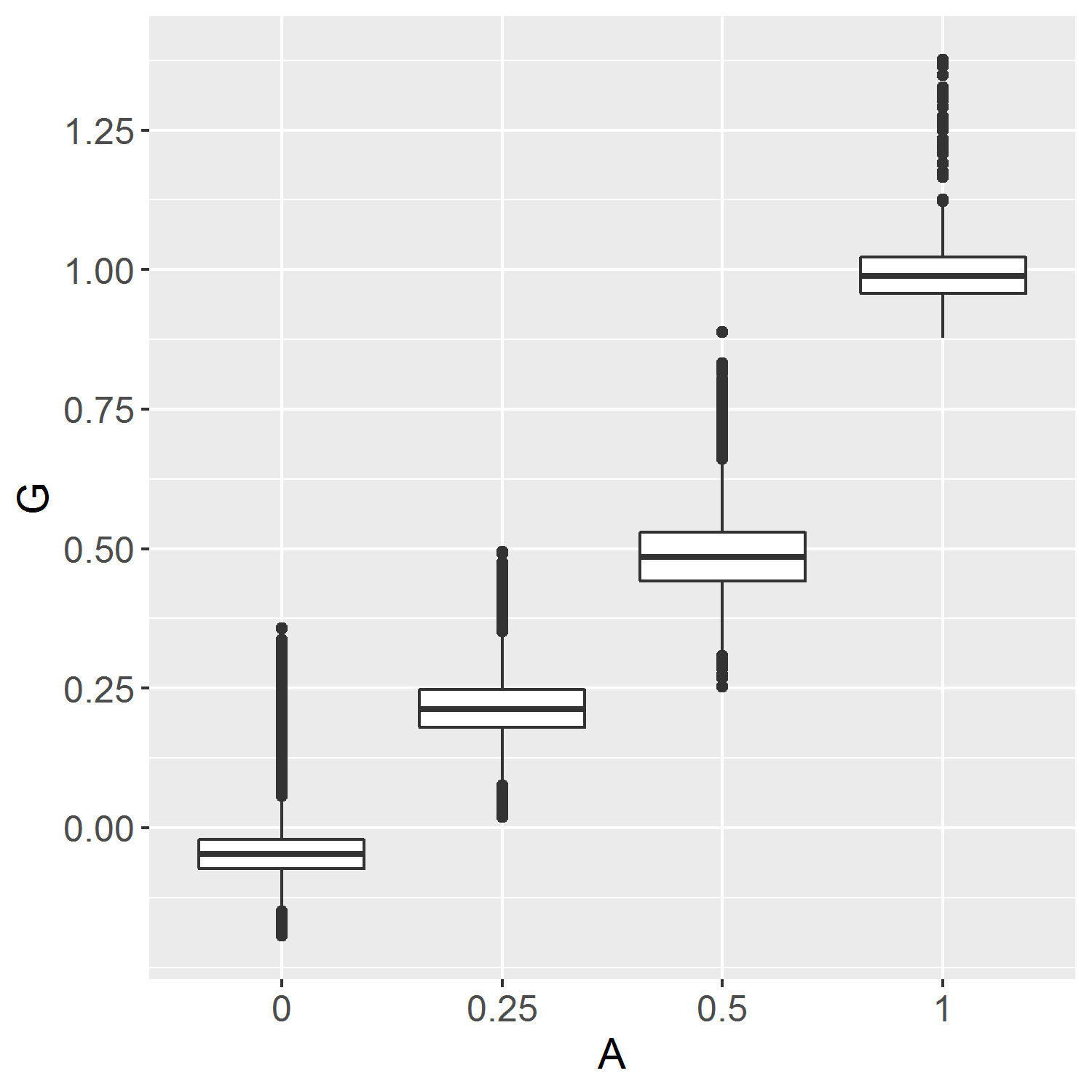


**Figure S6.** Comparison between the pedigree-based relationships matrix ($\boldsymbol{A}$) and the realized genomic relationship matrix (**G**) after removing nine miss-classified individuals (pedigree errors), two clones, and one outlier tree with high wood density. Large values of **G** within categories of $\boldsymbol{A}$ are mostly due to two inbred families (Fam_1 and Fam_2, see figure S7).


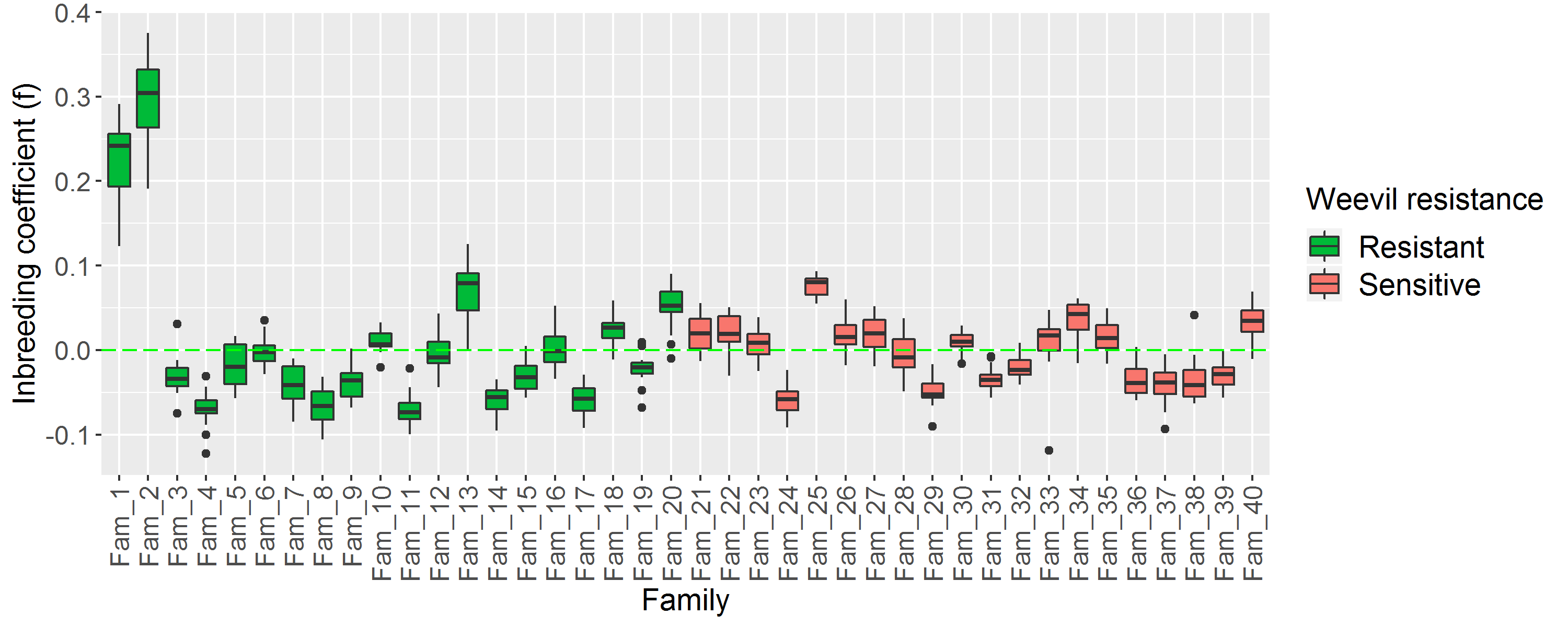
**Figure S7.** Realized inbreeding coefficient obtained from the diagonal elements of the realized genomic relationship matrix (**G**) minus 1. Resistant and sensitive families are colored in green and orange, respectively. Families Fam_1 and Fam_2 show inbreeding (f > 0.1, i.e. the parents crossed had recent common ancestors).

| (A) | 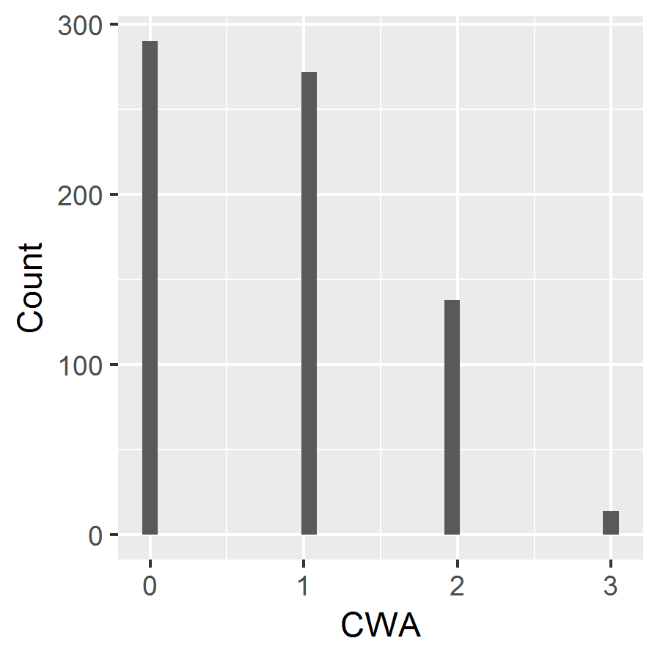 |
| --- | --- |
| (B) | 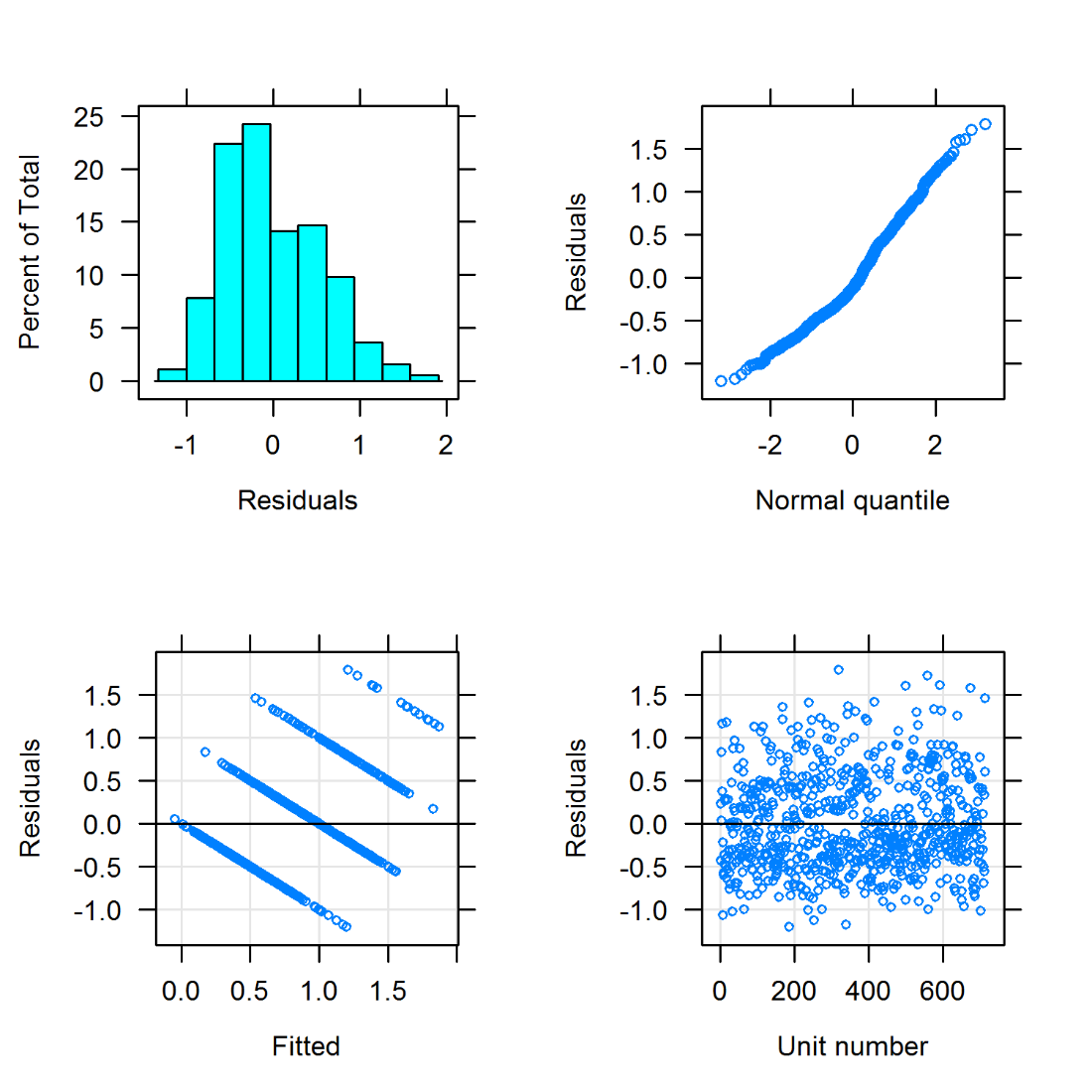 |

**Figure S8.** (A) Histogram of the cumulative number of weevil attacks (CWA). (B) Distribution of residuals for CWA modeled with GBLUP (equation [2] in manuscript), which assumed normality of errors.


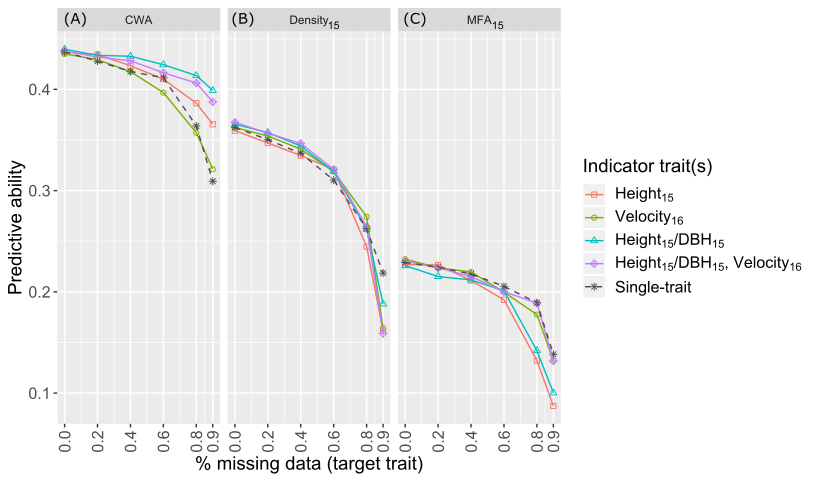


**Figure S9.** Predictive ability (PA) of GBLUP multi-trait genomic selection models for predicting the target trait: (A) the cumulative number of weevil attacks (CWA); (B) Density_15_; and (C) MFA_15_. The different colored lines represent different multi-trait models with different indicator traits. The dashed gray line is the single-trait GBLUP model. The percentage of missing phenotypic data for the focal trait in the training sets was varied from 0 to 90% (x-axis), while 100% of the training data was retained for the indicator traits.
