# Supplementary material for: Multi‐trait genomic selection for weevil resistance, growth, and wood quality in Norway spruce
Source: Evol Appl. 2019 Jun 20;13(1):76–94. doi: 10.1111/eva.12823 (PMC6935592; doi:10.1111/eva.12823)
Supplement: Supplementary file 2 [file EVA-13-76-s002.docx]

**Table S1.** Results of models including additive and dominance effects^1^: variance components, heritability, type-B genetic correlation estimates, and number of degrees of freedom using the ABLUP and GBLUP methods for the across-site analysis. Standard errors of estimates are in parentheses. This table shows that the estimated dominance variance $\hat{\sigma}_{d}^{2}$ was not significant for all traits under study.

|  | Velocity_16_ | Density_15_ | MFA_15_ | DBH_15_ | Height_15_ | Height_15_/DBH_15_ | CWA |
| --- | --- | --- | --- | --- | --- | --- | --- |
|  | (km/s*10²) | (kg/m³) | (mm) | (cm) | (cm) | — | (number of attacks) |
| **ABLUP** |  |  |  |  |  |  |  |
| $\hat{\sigma}_{b}^{2}$ | 0.0109 (0.0060) *** | 31.4 (20.0) *** | 0.00 (0.00) | 14.8 (10.0) *** | 474 (311) *** | 5.59 (3.40) *** | 0.0027 (0.0047) |
| $\hat{\sigma}_{a}^{2}$ | 0.0310 (0.0139) ** | 167.3 (93.9) * | 2.40 (1.91) | 0.0 (0.0) | 4570 (3179) | 38.54 (19.85) * | 0.2483 (0.1015) ** |
| $\hat{\sigma}_{sa}^{2}$ | 0.0000 (0.0000) | 26.6 (73.0) | 2.35 (2.11) | 0.0 (0.0) | 2405 (1949) | 0.00 (0.00) | 0.0000 (0.0000) |
| $\hat{\sigma}_{d}^{2}$ | 0.0087 (0.0158) | 5.96 (119.1) | 0.00 (0.00) | 0.0 (0.0) | 1821 (3329) | 10.77 (24.56) | 0.0504 (0.0865) |
| $\hat{\sigma}_{sd}^{2}$ | 0.0219 (0.0126) | 152.7 (155.3) | 1.57 (3.46) | 191.8 (57.6) ** | 3379 (3111) | 49.06 (19.59) * | 0.0380 (0.0572) |
| $\hat{\sigma}_{e}^{2}$ | 0.0333 (0.0124) | 352.7 (87.7) | 24.09 (3.06) | 209 (50) | 2599 (2125) | 15.42 (17.45s) | 0.2790 (0.0716) |
| $\hat{h}_{ind}^{2}$ | 0.33 (0.13) | 0.24 (0.12) | 0.08 (0.06) | 0.00 (0.00) | 0.31 (0.20) | 0.34 (0.15) | 0.40 (0.14) |
| $\hat{d}_{ind}^{2}$ | 0.09 (0.17) | 0.01 (0.17) | 0.00 (0.00) | 0.00 (0.00) | 0.12 (0.23) | 0.09 (0.22) | 0.08 (0.14) |
| $\hat{H}_{ind}^{2}$ | 0.42 (0.16) | 0.25 (0.14) | 0.08 (0.06) | 0.00 (0.00) | 0.43 (0.18) | 0.43 (0.19) | 0.49 (0.14) |
| $\hat{r}_{B ADD}$ | 1.00 (0.00) | 0.86 (0.36) | 0.51 (0.36) | 0.21 (0.00) | 0.66 (0.28) | 1.00 (0.00) | 1.00 (0.00) |
| $\hat{r}_{B DOM}$ | 0.29 (0.43) | 0.04 (0.75) | 0.00 (0.00) | 0.00 (0.00) | 0.35 (0.55) | 0.18 (0.36) | 0.57 (0.64) |
| nedf | 712 | 712 | 706 | 712 | 712 | 712 | 712 |
|  |  |  |  |  |  |  |  |
| **GBLUP** |  |  |  |  |  |  |  |
| $\hat{\sigma}_{b}^{2}$ | 0.0100 (0.0055) *** | 27.7 (18.2) *** | 0.00 (0.00) | 13.8 (9.6) ** | 401 (276) ** | 5.96 (3.61) *** | 0.0028 (0.0048) |
| $\hat{\sigma}_{a}^{2}$ | 0.0292 (0.0078) *** | 189.9 (57.8) ** | 1.90 (1.55) | 0.0 (0.0) | 2076 (1206) | 18.97 (8.96) * | 0.1551 (0.0471) *** |
| $\hat{\sigma}_{sa}^{2}$ | 0.0000 (0.0000) | 0.0 (0.0) | 2.30 (1.79) * | 22.9 (23.2) | 2044 (1269) * | 12.21 (9.40) | 0.0248 (0.0281) |
| $\hat{\sigma}_{d}^{2}$ | 0.0000 (0.0000) | 0.0 (0.0) | 0.00 (0.00) | 18.1 (35.4) | 1450 (1360) | 6.70 (9.47) | 0.0131 (0.0360) |
| $\hat{\sigma}_{sd}^{2}$ | 0.0225 (0.0076) ** | 149.2 (62.1) | 1.57 (2.55) | 76.4 (52.6) | 2378 (1795) | 13.22 (12.29) | 0.0000 (0.0000) |
| $\hat{\sigma}_{e}^{2}$ | 0.0399 (0.0072) | 369.5 (58.1) | 24.32 (2.56) | 281.4 (33.3) | 5647 (1080) | 53.95 (8.35) | 0.3846 (0.0406) |
| $\hat{h}_{ind}^{2}$ | 0.32 (0.07) | 0.27 (0.07) | 0.06 (0.05) | 0.00 (0.00) | 0.15 (0.09) | 0.18 (0.08) | 0.27 (0.07) |
| $\hat{d}_{ind}^{2}$ | 0.00 (0.00) | 0.00 (0.00) | 0.00 (0.00) | 0.05 (0.09) | 0.11 (0.10) | 0.06 (0.09) | 0.02 (0.06) |
| $\hat{H}_{ind}^{2}$ | 0.32 (0.07) | 0.27 (0.07) | 0.06 (0.05) | 0.05 (0.09) | 0.26 (0.10) | 0.24 (0.10) | 0.29 (0.08) |
| $\hat{r}_{B ADD}$ | 1.00 (0.00) | 1.00 (0.00) | 0.45 (0.34) | 0.00 (0.00) | 0.50 (0.26) | 0.61 (0.26) | 0.86 (0.15) |
| $\hat{r}_{B DOM}$ | 0.00 (0.00) | 0.00 (0.00) | 0.00 (0.00) | 0.19 (0.38) | 0.38 (0.36) | 0.34 (0.47) | 1.00 (0.00) |
| nedf | 712 | 712 | 706 | 712 | 712 | 712 | 712 |

^1^Dominance effects were added to equation [2]: $\boldsymbol{y}=\mu+\boldsymbol{X\beta}+ \boldsymbol{Z}_{\mathbf{1}}\boldsymbol{b}\left( \boldsymbol{s} \right)+\boldsymbol{Z}_{\mathbf{2}}\boldsymbol{a}+\boldsymbol{Z}_{\mathbf{3}}\boldsymbol{sa+}\boldsymbol{Z}_{\mathbf{4}}\boldsymbol{d+}\boldsymbol{Z}_{\mathbf{4}}\boldsymbol{sd}+\boldsymbol{e}$, where $\boldsymbol{d}$ is the random dominance genetic effect, with $\boldsymbol{d}\sim N\left( 0,\sigma_{d}^{2}\boldsymbol{A}_{\boldsymbol{Dom}} \right)$ for the ABLUP model and $\boldsymbol{d}\sim N\left( 0,\sigma_{d}^{2}\boldsymbol{G}_{\boldsymbol{Dom}} \right)$ for the GBLUP model; $\boldsymbol{sd}$ is the random interaction of site with dominance genetic effects, with $\boldsymbol{sd}\sim N(0,\sigma_{sd}^{2}\boldsymbol{I}_{\boldsymbol{s}}\boldsymbol{A}_{\boldsymbol{Dom}}$) for the ABLUP model and $\boldsymbol{sd}\sim N(0,\sigma_{sd}^{2}\boldsymbol{I}_{\boldsymbol{s}}\boldsymbol{G}_{\boldsymbol{Dom}}$) for the GBLUP model. $\boldsymbol{A}_{\boldsymbol{Dom}}$ is the dominance relationship matrix obtained from the pedigree using function makeD of the R package nadiv. $\boldsymbol{G}_{\boldsymbol{Dom}}$ is the realized dominance relationship matrix calculated following Vitezica et al (2013, *Genetics*, *195*(4), 1223–1230).

$\hat{\sigma}_{b}^{2}$ = block variance; $\hat{\sigma}_{a}^{2}$ = additive variance; $\hat{\sigma}_{sa}^{2}$ = site-by-additive interaction variance; $\hat{\sigma}_{d}^{2}$ = dominance variance; $\hat{\sigma}_{sd}^{2}$ = site-by-dominance interaction variance; $\hat{\sigma}_{e}^{2}$ = residual variance; $\hat{h}_{ind}^{2}$ = individual narrow-sense heritability calculated as $\hat{h}_{ind}^{2}={\hat{\sigma}_{a}^{2}}/{(\hat{\sigma}_{a}^{2}+\hat{\sigma}_{sa}^{2}+\hat{\sigma}_{d}^{2}+ \hat{\sigma}_{sd}^{2}+\hat{\sigma}_{e}^{2})}$; $\hat{d}_{ind}^{2}$ = proportion of phenotypic variance due to dominance calculated as $\hat{d}_{ind}^{2}={\hat{\sigma}_{d}^{2}}/{(\hat{\sigma}_{a}^{2}+\hat{\sigma}_{sa}^{2}+\hat{\sigma}_{d}^{2}+ \hat{\sigma}_{sd}^{2}+\hat{\sigma}_{e}^{2})}$; $\hat{H}_{ind}^{2}$ = individual broad-sense heritability calculated as $\hat{H}_{ind}^{2}={{(\hat{\sigma}}_{a}^{2}+\hat{\sigma}_{d}^{2})}/{(\hat{\sigma}_{a}^{2}+\hat{\sigma}_{sa}^{2}+\hat{\sigma}_{d}^{2}+ \hat{\sigma}_{sd}^{2}+\hat{\sigma}_{e}^{2})}$; $\hat{r}_{B (Add)}$ = type-B genetic additive correlation calculated as $\hat{r}_{B (Add)}={\hat{\sigma}_{a}^{2}}/{(\hat{\sigma}_{a}^{2}+\hat{\sigma}_{sa}^{2})}$; $\hat{r}_{B (Dom)}$ = type-B genetic dominance correlation $\hat{r}_{B (Dom)}={\hat{\sigma}_{d}^{2}}/{(\hat{\sigma}_{d}^{2}+\hat{\sigma}_{sd}^{2})}$; nedf = number of degrees of freedom.

Significance tests of variance components: * = *P* < 0.05; ** = *P* < 0.01; *** = *P* < 0.001.

**Table S2.** Fit of ABLUP and GBLUP models including additive effect only (A) and models including additive and dominance effects (A+D) as measured by the Akaike information criterion (AIC) and the Bayesian information criterion (BIC) for the across-site analysis.

|  | AIC | | | |  | BIC | | | |
| --- | --- | --- | --- | --- | --- | --- | --- | --- | --- |
|  | A | A+D | AIC Diff | Best model^1^ |  | A | A+D | BIC Diff | Best model^1^ |
| **ABLUP** |  |  |  |  |  |  |  |  |  |
| Velocity_16_ | -1037 | -1040 | 3 | AD |  | -1018 | -1013 | -6 | A |
| Density_15_ | 5334 | 5336 | -2 | — |  | 5353 | 5363 | -11 | A |
| MFA_15_ | 3110 | 3113 | -4 | A |  | 3128 | 3141 | -13 | A |
| DBH_15_ | 4992 | 4984 | 8 | AD |  | 5011 | 5012 | -1 | — |
| Height_15_ | 7411 | 7410 | 1 | — |  | 7429 | 7438 | -9 | A |
| Height_15_/DBH_15_ | 3977 | 3968 | 9 | AD |  | 3995 | 3995 | 0 | — |
| CWA | 263 | 266 | -2 | — |  | 281 | 293 | -12 | A |
|  |  |  |  |  |  |  |  |  |  |
| **GBLUP** |  |  |  |  |  |  |  |  |  |
| Velocity_16_ | -1053 | -1061 | 8 | AD |  | -1035 | -1034 | -1 | — |
| Density_15_ | 5335 | 5333 | 2 | — |  | 5353 | 5360 | -7 | A |
| MFA_15_ | 3107 | 3111 | -4 | A |  | 3126 | 3138 | -13 | A |
| DBH_15_ | 4995 | 4988 | 6 | AD |  | 5013 | 5016 | -3 | A |
| Height_15_ | 7428 | 7418 | 10 | AD |  | 7446 | 7446 | 1 | — |
| Height_15_/DBH_15_ | 3978 | 3976 | 2 | — |  | 3997 | 4004 | -7 | A |
| CWA | 264 | 268 | -4 | A |  | 283 | 296 | -13 | A |

^1^The best model was determined as the model with the smallest value of AIC or BIC, with ΔAIC > 2 or ΔBIC > 2.

**Table S3.** Comparison of ABLUP and GBLUP models fitting a unique residual variance across-site (Uniq)^1^ and models fitting a different residual variance for each site (Diff)^2^. The results reported are individual narrow-sense heritability ($\hat{h}_{ind}^{2}$) and type-B genetic correlation ($\hat{r}_{B}$) estimates, correlation of breeding values between both approaches (Corr. of BVs), fit of the models as measured by the Akaike information criterion (AIC) and the Bayesian information criterion (BIC). Standard errors of estimates are in parentheses.

|  | $\hat{h}_{ind}^{2}$ | |  | $\hat{r}_{B}$ | |  | Corr. of BVs |  | AIC | | | |  | BIC | | | |
| --- | --- | --- | --- | --- | --- | --- | --- | --- | --- | --- | --- | --- | --- | --- | --- | --- | --- |
|  | Uniq | Diff |  | Uniq | Diff |  |  |  | Uniq | Diff | AIC Diff | Best^3^ |  | Uniq | Diff | BIC Diff | Best^3^ |
| **ABLUP** |  |  |  |  |  |  |  |  |  |  |  |  |  |  |  |  |  |
| Velocity_16_ | 0.37 (0.12) ** | 0.28 (0.10) ** |  | 0.79 (0.15) | 0.79 (0.15) |  | 0.999 |  | -1037 | -1037 | 1 | — |  | -1018 | -1014 | -4 | Uniq |
| Density_15_ | 0.25 (0.11) * | 0.21 (0.10) * |  | 0.65 (0.20) * | 0.65 (0.20) * |  | 0.994 |  | 5334 | 5321 | 14 | Diff |  | 5353 | 5344 | 9 | Diff |
| MFA_15_ | 0.08 (0.06) | 0.05 (0.04) |  | 0.47 (0.32) * | 0.54 (0.35) |  | 0.992 |  | 3110 | 3081 | 29 | Diff |  | 3128 | 3104 | 24 | Diff |
| DBH_15_ | 0.00 (0.00) | 0.00 (0.06) |  | 0.00 (0.00) *** | 0.00 (0.39) *** |  | 1.000 |  | 4992 | 4994 | -2 | — |  | 5011 | 5017 | -7 | Uniq |
| Height_15_ | 0.47 (0.16) ** | 0.41 (0.16) ** |  | 0.65 (0.15) *** | 0.65 (0.15) *** |  | 1.000 |  | 7411 | 7413 | -2 | — |  | 7429 | 7436 | -7 | Uniq |
| Height_15_/DBH_15_ | 0.40 (0.14) ** | 0.35 (0.13) ** |  | 0.68 (0.16) ** | 0.69 (0.16) ** |  | 0.992 |  | 3977 | 3964 | 13 | Diff |  | 3995 | 3987 | 8 | Diff |
| CWA | 0.47 (0.12) *** | 0.38 (0.11) *** |  | 0.97 (0.08) | 0.97 (0.08) |  | 1.000 |  | 263 | 265 | -2 | — |  | 281 | 288 | -7 | Uniq |
|  |  |  |  |  |  |  |  |  |  |  |  |  |  |  |  |  |  |
| **GBLUP** |  |  |  |  |  |  |  |  |  |  |  |  |  |  |  |  |  |
| Velocity_16_ | 0.29 (0.08) *** | 0.22 (0.06) *** |  | 0.76 (0.16) | 0.75 (0.16) * |  | 0.999 |  | -1053 | -1055 | 2 | — |  | -1035 | -1032 | -3 | Uniq |
| Density_15_ | 0.26 (0.08) ** | 0.22 (0.07) ** |  | 0.76 (0.17) | 0.76 (0.16) |  | 0.994 |  | 5335 | 5321 | 14 | Diff |  | 5353 | 5344 | 9 | Diff |
| MFA_15_ | 0.06 (0.05) | 0.05 (0.04) |  | 0.43 (0.32) ** | 0.48 (0.33) * |  | 0.993 |  | 3107 | 3079 | 29 | Diff |  | 3126 | 3102 | 24 | Diff |
| DBH_15_ | 0.00 (0.00) | 0.00 (0.04) |  | 0.00 (0.00) ** | 0.00 (0.41) *** |  | 1.000 |  | 4995 | 4997 | -2 | — |  | 5013 | 5019 | -7 | Uniq |
| Height_15_ | 0.22 (0.08) ** | 0.17 (0.07) ** |  | 0.52 (0.17) *** | 0.52 (0.17) *** |  | 1.000 |  | 7428 | 7430 | -2 | — |  | 7446 | 7453 | -7 | Uniq |
| Height_15_/DBH_15_ | 0.20 (0.08) * | 0.16 (0.06) * |  | 0.56 (0.20) ** | 0.58 (0.20) ** |  | 0.996 |  | 3978 | 3966 | 13 | Diff |  | 3997 | 3989 | 8 | Diff |
| CWA | 0.27 (0.07) *** | 0.20 (0.06) *** |  | 0.86 (0.15) | 0.86 (0.15) |  | 1.000 |  | 264 | 266 | -2 | — |  | 283 | 289 | -7 | Uniq |

^1^The model fitted is described in equation [2] in the manuscript, with $\boldsymbol{e}\sim N(0,\sigma_{e}^{2}\boldsymbol{I}_{\boldsymbol{e}}$).

^2^The model fitted is described in equation [2] in the manuscript, with $\boldsymbol{e}\sim N\left( 0,\sigma_{e1}^{2}\boldsymbol{I}_{\boldsymbol{e}\boldsymbol{1}} \oplus\sigma_{e2}^{2}\boldsymbol{I}_{\boldsymbol{e}\boldsymbol{2}} \right)$, where ⊕ is the direct sum.

^3^The best model was determined as the model with the smallest value of AIC or BIC, with ΔAIC > 2 or ΔBIC > 2.

**Table S4.** Variance components estimated using the single-trait ABLUP and GBLUP methods for the across-site analysis^1^. Standard errors of estimates are in parentheses.

|  | Velocity_16_ | Density_15_ | MFA_15_ | DBH_15_ | Height_15_ | Height_15_/DBH_15_ | CWA |
| --- | --- | --- | --- | --- | --- | --- | --- |
|  | (km/s*10²) | (kg/m³) | (degrees) | (mm) | (cm) | — | (number of attacks) |
| **ABLUP^2^** |  |  |  |  |  |  |  |
| $\hat{\sigma}_{b}^{2}$ | 0.0110 (0.0060) *** | 30.7 (19.7) *** | 0.00 (0.00) | 14.7 (10.1) ** | 477 (314) *** | 5.26 (3.26) *** | 0.0026 (0.0047) |
| $\hat{\sigma}_{a}^{2}$ | 0.0351 (0.0135) ** | 176.4 (84.4) * | 2.40 (1.90) | 0.0 (0.0) | 7424 (3055) ** | 47.57 (19.45) ** | 0.2934 (0.0965) *** |
| $\hat{\sigma}_{sa}^{2}$ | 0.0093 (0.0070) | 93.2 (57.0) * | 2.69 (1.91) * | 95.4 (36.8) *** | 4005 (1702) *** | 22.59 (11.69) ** | 0.0093 (0.0249) |
| $\hat{\sigma}_{e}^{2}$ | 0.0518 (0.0082) | 444.3 (55.4) | 25.28 (1.90) | 318.3 (29.8) | 4490 (1662) | 48.31 (11.12) | 0.3241 (0.0565) |
|  |  |  |  |  |  |  |  |
| **GBLUP^2^** |  |  |  |  |  |  |  |
| $\hat{\sigma}_{b}^{2}$ | 0.0101 (0.0056) *** | 27.7 (18.2) *** | 0.00 (0.00) | 14.7 (10.1) ** | 465 (311) *** | 5.58 (3.43) *** | 0.0027 (0.0048) |
| $\hat{\sigma}_{a}^{2}$ | 0.0262 (0.0081) *** | 186.5 (61.8) ** | 1.91 (1.55) | 0.00 (0.00) | 3102 (1227) ** | 21.14 (8.67) * | 0.1584 (0.0457) *** |
| $\hat{\sigma}_{sa}^{2}$ | 0.0084 (0.0059) | 57.6 (43.3) | 2.55 (1.73) ** | 61.0 (25.1) ** | 2843 (1187) *** | 16.78 (8.71) ** | 0.0256 (0.0284) |
| $\hat{\sigma}_{e}^{2}$ | 0.0565 (0.0054) | 467.2 (42.5) | 25.6 (1.77) | 342.9 (25.4) | 7992 (800) | 67.66 (6.07) | 0.3935 (0.0332) |

^1^The model fitted is described in equation [2] in the manuscript. $\hat{\sigma}_{b}^{2}$ = block variance; $\hat{\sigma}_{a}^{2}$ = additive variance; $\hat{\sigma}_{sa}^{2}$ = site-by-additive interaction variance; $\hat{\sigma}_{e}^{2}$ = residual variance.

^2^Significance tests for variance components: * = *P* < 0.05; ** = *P* < 0.01; *** = *P* < 0.001.

**Table S5.** Site GPI: phenotypic ($\hat{r}_{p}$, above diagonal) and genetic correlations ($\hat{r}_{a}$, below diagonal) between traits calculated with the ABLUP method^1^. Diagonal elements indicate the single-site narrow-sense heritability ($\hat{h}_{ind ss}^{2}$) for each trait. Standard errors of estimates are in parentheses. Genetic and phenotypic correlations were tested for significance^2^. For $\hat{h}_{ind ss}^{2}$, the significance of the additive variance component is shown^2^.

| Trait | Velocity_16_ | Density_15_ | MFA_15_ | DBH_15_ | Height_15_ | Height_15_/DBH_15_ | CWA |
| --- | --- | --- | --- | --- | --- | --- | --- |
| Velocity_16_ | 0.55 (0.14) *** | 0.34 (0.07) *** | -0.1 (0.05) | -0.17 (0.07) ** | 0.34 (0.07) *** | 0.47 (0.06) *** | -0.27 (0.07) ** |
| Density_15_ | 0.6 (0.16) ** | 0.56 (0.15) *** | -0.04 (0.06) | -0.47 (0.05) *** | -0.04 (0.09) | 0.39 (0.07) *** | -0.14 (0.08) |
| MFA_15_ | -0.04 (0.42) | -0.37 (0.39) | 0.06 (0.05) | 0.02 (0.05) | 0.03 (0.06) | -0.01 (0.06) | 0.03 (0.06) |
| DBH_15_ | -0.18 (0.28) | -0.51 (0.22) | -0.22 (0.51) | 0.28 (0.13) ** | 0.38 (0.06) *** | -0.53 (0.05) *** | 0.15 (0.07) |
| Height_15_ | 0.65 (0.16) ** | -0.01 (0.24) | 0.45 (0.36) | 0.34 (0.24) | 0.89 (0.16) *** | 0.57 (0.06) *** | -0.53 (0.06) *** |
| Height_15/_DBH_15_ | 0.78 (0.11) *** | 0.39 (0.2) | 0.45 (0.37) | -0.31 (0.24) | 0.78 (0.11) *** | 0.72 (0.16) *** | -0.6 (0.05) *** |
| CWA | -0.64 (0.16) ** | -0.27 (0.23) | -0.22 (0.42) | 0.51 (0.23) | -0.73 (0.12) *** | -0.98 (0.03) *** | 0.71 (0.16) *** |

^1^The model fitted is described in equation [5] in the manuscript.

^2^Significance tests: * = *P* < 0.05; ** = *P* < 0.01; *** = *P* < 0.001.

**Table S6.** Site STM: phenotypic ($\hat{r}_{p}$, above diagonal) and genetic correlations ($\hat{r}_{a}$, below diagonal) between traits calculated with the ABLUP method^1^. Diagonal elements indicate the single-site narrow-sense heritability ($\hat{h}_{ind ss}^{2}$) for each trait. Standard errors of estimates are in parentheses. Genetic and phenotypic correlations were tested for significance^2^. For $\hat{h}_{ind ss}^{2}$, the significance of the additive variance component is shown^2^.

| Trait | Velocity_16_ | Density_15_ | MFA_15_ | DBH_15_ | Height_15_ | Height_15_/DBH_15_ | CWA |
| --- | --- | --- | --- | --- | --- | --- | --- |
| Velocity_16_ | 0.45 (0.14) *** | 0.26 (0.08) *** | -0.33 (0.06) *** | -0.20 (0.08) * | 0.22 (0.08) * | 0.44 (0.06) *** | -0.23 (0.07) ** |
| Density_15_ | 0.03 (0.32) | 0.23 (0.10) *** | 0.14 (0.06) | -0.42 (0.06) *** | -0.14 (0.07) ** | 0.40 (0.06) *** | -0.23 (0.06) *** |
| MFA_15_ | -0.83 (0.13) *** | 0.22 (0.33) | 0.23 (0.10) *** | -0.05 (0.06) | -0.14 (0.06) | -0.05 (0.07) | -0.02 (0.06) |
| DBH_15_ | -0.49 (0.29) | 0.01 (0.39) | 0.66 (0.28) | 0.19 (0.11) * | 0.61 (0.05) *** | -0.69 (0.04) *** | 0.25 (0.06) *** |
| Height_15_ | 0.53 (0.24) | 0.16 (0.33) | -0.16 (0.32) | 0.18 (0.35) | 0.35 (0.14) *** | 0.14 (0.08) | -0.23 (0.06) ** |
| Height_15_/DBH_15_ | 0.79 (0.13) ** | 0.19 (0.30) | -0.69 (0.21) * | -0.64 (0.20) * | 0.67 (0.21) * | 0.51 (0.15) *** | -0.52 (0.05) *** |
| CWA | -0.69 (0.20) * | -0.05 (0.33) | 0.18 (0.32) | 0.44 (0.30) | -0.5 (0.26) | -0.74 (0.15) ** | 0.28 (0.11) *** |

^1^The model fitted is described in equation [5] in the manuscript.

^2^Significance tests: * = *P* < 0.05; ** = *P* < 0.01; *** = *P* < 0.001.

**Table S7.** Predictive accuracy (PACC) of GBLUP multi-trait genomic selection models^1^ for predicting a target trait (CWA, Density_16_ or MFA_15_) when coupled with genetically correlated indicator traits (Velocity_16_, Height_15_/DBH_15_, or Height_15_/DBH_15_). Results of multi-trait models are compared with the single-trait GBLUP model^2^. The percentage of missing phenotypic data for the target trait in the training sets was varied from 0 to 90%, while 100% of the training data was retained for the indicator traits. Standard errors of estimates are in parentheses.

|  |  | Single-trait model |  | Multi-trait models: Indicator trait(s) | | | |
| --- | --- | --- | --- | --- | --- | --- | --- |
| Focal trait | missing data (%) |  |  | Height_15_ | Velocity_16_ | Height_15_/DBH_15_ | Height_15_/DBH_15_ and Velocity_16_ |
| CWA |  |  |  |  |  |  |  |
|  | 0 | 0.83 (0.17) |  | 0.83 (0.17) | 0.83 (0.17) | 0.84 (0.16) | 0.84 (0.16) |
|  | 20 | 0.82 (0.16) |  | 0.83 (0.17) | 0.82 (0.16) | 0.83 (0.16) | 0.82 (0.17) |
|  | 40 | 0.80 (0.17) |  | 0.81 (0.19) | 0.80 (0.17) | 0.83 (0.16) | 0.82 (0.16) |
|  | 60 | 0.79 (0.18) |  | 0.78 (0.18) | 0.76 (0.17) | 0.81 (0.15) | 0.80 (0.15) |
|  | 80 | 0.69 (0.18) |  | 0.74 (0.19) | 0.68 (0.18) | 0.79 (0.15) | 0.78 (0.15) |
|  | 90 | 0.59 (0.20) |  | 0.70 (0.18) | 0.61 (0.18) | 0.76 (0.16) | 0.74 (0.17) |
|  |  |  |  |  |  |  |  |
| Density_15_ |  |  |  |  |  |  |  |
|  | 0 | 0.71 (0.18) |  | 0.70 (0.18) | 0.71 (0.18) | 0.71 (0.18) | 0.72 (0.18) |
|  | 20 | 0.68 (0.19) |  | 0.68 (0.19) | 0.69 (0.19) | 0.70 (0.18) | 0.70 (0.18) |
|  | 40 | 0.66 (0.20) |  | 0.65 (0.19) | 0.67 (0.18) | 0.67 (0.19) | 0.68 (0.17) |
|  | 60 | 0.61 (0.20) |  | 0.63 (0.22) | 0.62 (0.18) | 0.62 (0.19) | 0.63 (0.20) |
|  | 80 | 0.51 (0.21) |  | 0.48 (0.22) | 0.54 (0.22) | 0.51 (0.23) | 0.52 (0.22) |
|  | 90 | 0.43 (0.23) |  | 0.32 (0.27) | 0.32 (0.27) | 0.37 (0.27) | 0.31 (0.30) |
|  |  |  |  |  |  |  |  |
| MFA_15_ |  |  |  |  |  |  |  |
|  | 0 | 0.91 (0.44) |  | 0.90 (0.43) | 0.92 (0.40) | 0.90 (0.43) | 0.91 (0.39) |
|  | 20 | 0.89 (0.42) |  | 0.90 (0.43) | 0.89 (0.39) | 0.85 (0.44) | 0.89 (0.37) |
|  | 40 | 0.86 (0.44) |  | 0.84 (0.44) | 0.87 (0.38) | 0.84 (0.43) | 0.85 (0.40) |
|  | 60 | 0.82 (0.46) |  | 0.76 (0.44) | 0.79 (0.40) | 0.80 (0.45) | 0.79 (0.39) |
|  | 80 | 0.75 (0.40) |  | 0.52 (0.48) | 0.70 (0.39) | 0.56 (0.50) | 0.75 (0.41) |
|  | 90 | 0.55 (0.55) |  | 0.35 (0.56) | 0.52 (0.58) | 0.40 (0.50) | 0.52 (0.49) |

^1^The multi-trait GBLUP model fitted is described in equation [9] in the manuscript.

^2^The single-trait GBLUP model fitted is described in equation [11] in Appendix S2.

**Table S8.** Realized genetic gains when selecting the top 5% trees for each trait separately using the single-trait ABLUP and GBLUP method (across-site analysis). Gains are expressed as a percentage of the phenotypic mean. A positive percentage indicates an improvement in the value of the trait.

|  | Velocity_16_ | Density_15_ | MFA_15_^1^ | DBH_15_ | Height_15_ | Height_15_/DBH_15_ | CWA^1^ |  |
| --- | --- | --- | --- | --- | --- | --- | --- | --- |
|  | (%) | (%) | (%) | (%) | (%) | (%) | (%) |  |
| **ABLUP** | 7.27 | 4.07 | 7.71 | 0.00 | 14.18 | 14.51 | 77.59 |  |
| **GBLUP** | 7.11 | 5.05 | 9.09 | 0.00 | 9.05 | 11.23 | 65.85 |  |

^1^For MFA and CWA, an improvement (positive percentage) is associated with a decreasing value of the trait (i.e. a reduction of the microfibril angle and a reduction of the cumulative number of weevil attacks respectively).

**Table S9.** Posterior mean of estimated proportion of marker (π) having an effect in BayesCπ

| Trait | π | Standard error |
| --- | --- | --- |
| Velocity_16_ | 0.52 | 0.12 |
| Density_15_ | 0.46 | 0.14 |
| MFA_15_ | 0.41 | 0.14 |
| DBH_15_ | 0.32 | 0.13 |
| Height_15_ | 0.52 | 0.12 |
| Height_15_/DBH_15_ | 0.47 | 0.11 |
| CWA | 0.53 | 0.12 |

**Table S10.** Predictive ability (PA) and accuracy (PACC) of single-trait GBLUP models that 1) fitted GxE and used the unadjusted phenotype as the response variable (***y***)^1^; or 2) did not fit GxE and used the adjusted phenotype for block and site effects as a response variable ($\boldsymbol{y}^{\boldsymbol{*}}$)^2^. Only the traits that were modeled as focal traits in multi-trait GS models were tested. The modeling was done with 0% missing data in the training set. Standard errors of estimates are in parentheses.

|  | PA | |  | PACC | |
| --- | --- | --- | --- | --- | --- |
| Trait | ***y*** and GxE fitted | ***yadjust*** and no GxE fitted |  | ***y*** and GxE fitted | ***yadjust*** and no GxE fitted |
| CWA | 0.44 (0.09) | 0.44 (0.09) |  | 0.83 (0.17) | 0.83 (0.17) |
| Density_15_ | 0.36 (0.09) | 0.36 (0.09) |  | 0.71 (0.18) | 0.71 (0.18) |
| MFA_15_ | 0.23 (0.11) | 0.23 (0.11) |  | 0.91 (0.43) | 0.91 (0.44) |

^1^The model fitted is described in equation [2] in the manuscript.

^2^The model fitted is described in equation [11] in Appendix S2.
