# Supplementary material for: Multi‐trait genomic selection for weevil resistance, growth, and wood quality in Norway spruce
Source: Evol Appl. 2019 Jun 20;13(1):76–94. doi: 10.1111/eva.12823 (PMC6935592; doi:10.1111/eva.12823)
Supplement: Supplementary file 3 [file EVA-13-76-s003.docx]

**APPENDIX S1: Priors used for fitting the** **Bayesian ridge regression (BRR) and BayesCπ models**

To account for different distributions of marker effects, the prior for $\boldsymbol{a}_{\boldsymbol{m}}$ in equation [8] changes depending on the method (BRR or BayesCπ). BRR is a Bayesian version of ridge regression, in which marker effects are normally distributed (*i.e.* Gaussian prior) and have identical variance ($\boldsymbol{a}_{\boldsymbol{m}}\sim N(0,\sigma_{m}^{2}\boldsymbol{I}_{\boldsymbol{m}}$)). Variance components $\sigma_{b}^{2}$, $\sigma_{m}^{2}$, and $\sigma_{e}^{2}$ were assigned a scaled inverted $\chi^{2}$ distribution with $df$ degrees of freedom and scale parameter $S$ (${\sigma^{2}\sim\chi}^{-2}(df,S)$). In BRR, all marker effects are shrunk to a similar extent (*i.e.* all markers have a non-zero effect), and so this method is appropriate for traits controlled by a large number of genes with small effects.

BayesCπ takes into account that a number of genes have no effect on the trait of interest. It induces shrinkage of marker effects so that a proportion π of markers have an effect and a proportion (1 - π) are shrunk toward zero. This is modeled by assigning a prior for marker effects ($\boldsymbol{a}_{\boldsymbol{m}}$) that is a mixture of a point of mass at zero and a Gaussian slab (Habier et al., 2011). The parameter π is treated as unknown and is assigned a Beta prior $\pi\sim Beta\left( p_{0},\pi_{0} \right)$, with $p_{0}$ > 0 and $\pi_{0}\in[0,1]$.

We used the default starting parameters provided by BGLR. The convergence of BRR and BayesCπ models was verified by running 2 MCMC chains for each model and by using Gelman-Rubin diagnostic plots (shrink factor < 1.1) implemented in the R package coda (Plummer et al., 2006).

**APPENDIX S2: Comparisons between multi-trait and single-trait GBLUP models**

To facilitate direct comparisons of predictive ability and predictive accuracy, the multi-trait models (equation [9]) were compared with the equivalent single-trait GBLUP models fitted for each target trait. As for the multi-trait models, phenotypes were first adjusted for block and site effects ($\boldsymbol{y}^{\boldsymbol{*}}$) by taking the residuals ($\boldsymbol{e}$) of a model that included a fixed site effect ($\boldsymbol{s}$) and a random block within site effect ($\boldsymbol{b}\left( \boldsymbol{s} \right)$): $\boldsymbol{y}=\mu+ \boldsymbol{Xs}+ \boldsymbol{Zb}\left( \boldsymbol{s} \right)+\boldsymbol{e}$. Then, the following single-trait GBLUP model was fitted:

[11] $\boldsymbol{y}^{\boldsymbol{*}}=\mu+ \boldsymbol{Za}+\boldsymbol{e}$

Where $\boldsymbol{y}^{\boldsymbol{*}}$ is the adjusted phenotype**,** $\mu$ is the overall mean; $\boldsymbol{a}$ is the random additive genetic effect, with $\boldsymbol{a}\sim N\left( 0,\sigma_{a}^{2}\boldsymbol{G} \right)$; and $\boldsymbol{e}$ is the residual term, with $\boldsymbol{e}\sim N(0,\sigma_{e}^{2}\boldsymbol{I}_{\boldsymbol{e}}$).

**APPENDIX REFERENCES**

Habier, D., Fernando, R. L., Kizilkaya, K., and Garrick, D. J. (2011). Extension of the Bayesian alphabet for genomic selection. *BMC Bioinformatics* 12, 186. doi:10.1186/1471-2105-12-186.

Plummer, M., Best, N., Cowles, K., and Vines, K. (2006). CODA: convergence diagnosis and output analysis for MCMC. *R News* 6, 7–11.
